# Role of Iodine-Assisted Aerosol Particle Formation in Antarctica

**DOI:** 10.1021/acs.est.3c09103

**Published:** 2024-04-16

**Authors:** Carlton Xavier, Robin Wollesen de jonge, Tuija Jokinen, Lisa Beck, Mikko Sipilä, Tinja Olenius, Pontus Roldin

**Affiliations:** † Department of Physics, Lund University, Professorsgatan 1, Lund SE-22363, Sweden; ‡ Swedish Meteorological and Hydrological Institute (SMHI), Norrköping SE-60176, Sweden; § Institute for Atmospheric and Earth System Research (INAR)/Physics, Faculty of Science, University of Helsinki, P.O. Box 64, Helsinki 00014, Finland; ∥ Climate & Atmosphere Research Centre (CARE-C), The Cyprus Institute, P.O. Box 27456, Nicosia 1645, Cyprus; ⊥ Institute for Atmospheric and Environmental Sciences, Goethe University Frankfurt, Frankfurt am Main 60438, Germany; # Swedish Environmental Research Institute IVL, Malmö SE-21119, Sweden

**Keywords:** secondary aerosols, modeling, iodic acid, new particle formation, Southern Ocean

## Abstract

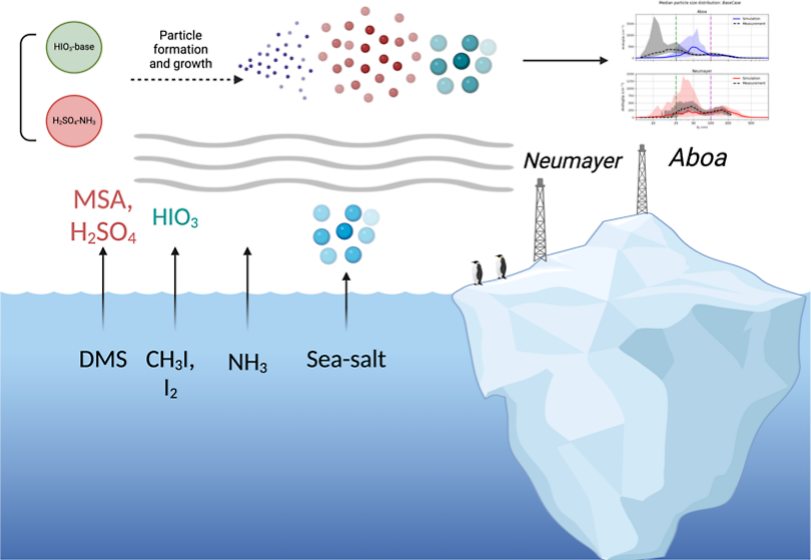

New particle formation via the ion-mediated sulfuric acid and ammonia molecular clustering mechanism remains the most widely observed and experimentally verified pathway. Recent laboratory and molecular level observations indicate iodine-driven nucleation as a potentially important source of new particles, especially in coastal areas. In this study, we assess the role of iodine species in particle formation using the best available molecular thermochemistry data and coupled to a detailed 1-d column model which is run along air mass trajectories over the Southern Ocean and the coast of Antarctica. In the air masses traversing the open ocean, ion-mediated SA-NH_3_ clustering appears insufficient to explain the observed particle size distribution, wherein the simulated Aitken mode is lacking. Including the iodine-assisted particle formation improves the modeled Aitken mode representation with an increase in the number of freshly formed particles. This implies that more particles survive and grow to Aitken mode sizes via condensation of gaseous precursors and heterogeneous reactions. Under certain meteorological conditions, iodine-assisted particle formation can increase cloud condensation nuclei concentrations by 20%–100%.

## Abstract 2

The impact of iodine-assisted particle formation in the Southern Ocean marine air masses is assessed. The study reports that iodine-assisted particle formation can play a significant role over open oceans, with potential implications to CCN concentrations.

## 1 Introduction

The impact of atmospheric aerosols on Earth’s climate via perturbations in radiative balance (direct effect) and cloud microphysics (indirect effect) is still relatively poorly understood and uncertain.^1,2^ Efforts in understanding the sources, chemical composition, and the physical properties of atmospheric aerosols are pivotal in reducing the uncertainty associated with both aerosol direct and indirect effects.^1,3−5^ Characterizing the atmospheric processes that govern the formation and growth of aerosols from natural sources in regions less impacted by anthropogenic influence is a basis for assessing the preindustrial atmosphere.^6^

Remote polar regions, such as Antarctica, are relatively less influenced by anthropogenic emissions^3,7,8^ and are therefore an ideal site to investigate the role of natural precursors on the formation and growth of secondary aerosols. In such pristine environments, new particle formation (NPF) is central for the formation of cloud condensation nuclei (CCN).^3,8,9^ Since Antarctica is surrounded by the Southern Ocean (SO), the marine environment plays an important role in NPF and in the subsequent growth of the freshly formed aerosols to larger sizes.^8^

Both models and observations suggest that secondary aerosol formation, both via NPF and growth, contributes more to CCN particles than primary marine aerosols such as sea-spray aerosols.^2,8−12^ NPF has been observed over both marine and coastal regions surrounding the Antarctic plateau^2,3,8,13^ as well as inland Antarctic measurement sites during the austral summer.^7,9,14^ Measurements indicate that secondary aerosol formation in Antarctica is well correlated with the oxidation products of oceanic emissions of dimethyl sulfide (DMS), namely, sulfuric acid (H_2_SO_4_; SA) and methanesulfonic acid (CH_3_SO_3_H; MSA).^9,15,16^ These DMS oxidation products can either directly cluster with various base molecules such as ammonia (NH_3_)^9,17^ and amines, for e.g., dimethylamine [(CH_3_)_2_NH; DMA]^13^ or possibly organics,^18−20^ to form new particles or condense together with bases onto pre-existing particles. The participation of ions in ion-mediated nucleation involving precursor vapors such as SA-NH_3_ and SA-DMA has also been documented.^9,17,21^

Although iodic acid (HIO_3_; ) IA is expected to contribute to NPF in marine and polar environments,^15,22^ its gas-phase chemical formation pathway is not fully resolved. Recent Cosmics Leaving OUtdoor Droplets (CLOUD) chamber experiments have shed light on a crucial pathway involving the ozonolysis of iodooxy hypoiodite (IOIO) which results in the formation of gas-phase IA.^23^ The sea-ice region and oceans, including snow and/or algae, diatoms and phytoplankton emissions in seawater surrounding the Antarctica Peninsula, are potential sources of IA emissions,^24,25^ which in the presence of stabilizers such as NH_3_, DMA, MSA, or iodous acid (HIO_2_) can initiate NPF.^26−28^ Earlier measurements around Halley, Neumayer, and the Weddell Sea suggested that iodine compounds originating near sea ice zones can participate in NPF. These newly formed particles can survive long enough to grow to CCN-relevant sizes.^29,30^

In this work, we model the secondary aerosol formation along the air masses arriving at two Antarctic research stations, Neumayer II (70.66° S, 8.27° W, 12th–19th January, 2019) and Aboa (73° 03′S, 13° 41′W, 7th–9th January, 2015), using a detailed aerosol and gas-phase process model coupled to an explicit multicompound molecular cluster dynamics model. The simulations performed in this work employ different multicomponent molecular cluster chemistries to assess the role of different clustering mechanisms in NPF. The roles of well-established and potential NPF clustering systems in the Southern marine environments and Antarctic continent are analyzed, namely, SA-NH_3_,^9,13^ SA-DMA,^3,13^ and IA-HIO_2_/DMA. Specifically, the contribution of IA with various stabilizers (HIO_2_/DMA) to NPF is explored, indicating an important role of such chemistries in secondary aerosol formation in marine polar regions.

## 2 Methods

### 2.1 Modeling Framework

The Aerosol Dynamics, gas- and particle-phase CHEMistry and radiative transfer model (ADCHEM),^31,32^ a 1-D column model, using 40 vertical layers (logarithmically spaced) extending up to ∼2600 m was run as a Lagrangian model along air mass trajectories arriving at the measurement station (Aboa and Neumayer) every third hour. A detailed description of the model setup and inputs (FLEXPART and ADCHEM) can be found in the Supporting Information (Model framework). The air mass trajectories and potential emission sensitivity fields were generated using FLEXPART v10.4.^33,34^ The widely used explicit chemical scheme detailing the gas-phase tropospheric reactions of volatile organic compounds, the Master chemical mechanism^35,36^ coupled to a comprehensive DMS and halogen multiphase oxidation chemistry scheme,^37^ was employed in this work. The chemistry scheme was further updated to include additional reactions which enabled the formation of gas-phase IA via the ozonolysis and subsequent hydrolysis of IOIO and its reaction products.^23^

In this work, we assumed cloud supersaturation (*S*_c_) of 0.5% in regions with liquid water content >0.01 g per m^3^ according to the ERA5 reanalysis meteorological data. ADCHEM combines an aerosol module which treats condensation/evaporation gases to/from particles and Brownian coagulation of aerosols with the molecular cluster dynamics module ClusterIn^38^ to describe the dynamics of the entire gas–cluster–aerosol system. ClusterIn explicitly simulates the temporal evolution of the cluster concentrations and the number and composition of the newly formed aerosol particles, gas–cluster partitioning, and cluster–aerosol coagulation by direct coupling to the aerosol dynamics module. This method was applied to circumvent possible artifacts that may arise from applying common simplifications to particle formation dynamics, enabling improved assessment of the relative importance of different clustering pathways to atmospheric NPF.^38^

The initial clustering processes were simulated by ClusterIn considering all possible collisions and evaporations among the clusters and/or vapor molecules (for more details, see ref (39)). The most important model parameters affecting the simulated cluster concentrations and thereby particle formation rates were the cluster evaporation rates, derived from molecular cluster thermochemistry data used as input in ClusterIn. To evaluate the role of different clustering species to NPF, we applied the most recent available data sets for molecular cluster thermochemistry, using combinations of SA, NH_3_, DMA, IA, and HIO_2_. Specifically, we applied ion-mediated SA-NH_3_^40^ and SA-DMA,^21^ pathways (ionization rate of 2 cm^–3^s^–1^) and neutral HIO_3_–HIO_2_^28^ (IA-HIO_2_), and HIO_3_-DMA^27^ (IA-DMA). For more details about the quantum chemical level of theory used in the data sets, please refer to the Supporting Information (Model framework).

To assess the role of IA-assisted particle formation for CCN concentrations, we used an adiabatic cloud parcel model.^31^ The activation of cloud droplets was calculated for 3 different updraft velocities *w* = 0.1 m s^–1^, *w* = 0.6 m s^–1^, and *w* = 1.0 m s^–1^, using the simulated size distribution, size-resolved chemical compositions, and gas-phase concentrations of SO_2_, H_2_O_2_, NH_3_, HNO_3_, SA, and HCl and cloud water-vapor supersaturations. The aerosol particles in the CCN size ranges were activated to cloud droplets, when relative humidity exceeded 100%, as the air parcels were transported upward to a maximum height of 160 m, subjected to the specific updraft velocity.

### 2.2 Sensitivity Tests

Initial particle formation at Aboa has been experimentally shown to be driven by ion-mediated SA-NH_3_ clustering.^9^ The role of SA-DMA, alongside SA-NH_3_ to particle formation, was also confirmed at the Marambio station by Quéléver et al., 2022. Here, we performed simulations using the molecular cluster thermochemical data using combinations of SA, NH_3_, DMA, IA, and HIO_2_. *BaseCase* refers to simulations including 3 clustering mechanisms, SA-NH_3_, SA-DMA, and IA-HIO_2_ simultaneously, to simulate secondary aerosol formation. The *BaseCase* simulations also included additional IA gas-phase reactions outlined by Finkenzeller et al., 2022. Comparison simulations *AN-AD* using only SA-NH_3_ and SA-DMA clustering systems were performed to assess the contribution of the IA-HIO_2_ clustering system to NPF. To assess the effects of IA-assisted NPF using different stabilizing species, another sensitivity test, *AN-AD-ID*, using the IA-DMA cluster thermochemistry data was performed. It should be noted that the IA-DMA cluster thermochemistry involves single-point energy calculations by the RICC2 method (Supporting Information), which has a general tendency to overpredict the cluster stabilities, resulting in higher formation rates. This implies that quantitative assessments of the relative importance or contribution of either IA-HIO_2_ (DLPNO-based cluster chemistries) or IA-DMA to iodine-assisted particle formation cannot be performed. The IA*-scaling* sensitivity tests were performed to study how well the simulated gas-phase IA concentrations compare to the measured values when omitting the IA gas-phase reactions based on Finkenzeller et al., 2022 (see reactions R1 and R2 in the Supporting Information). It must be noted that the formation pathway for HIO_2_ is still largely unknown, and this has a profound influence on the predicted formation rates from IA-HIO_2_ since this clustering pathway is sensitive to the concentrations of HIO_2_ (see Figure S6–S8 and discussion thereafter). Table 1 summarizes the different sensitivity tests performed in this work.

**Table 1:** Model Runs Performed in This Study, Including *BaseCase* and Sensitivity Tests, to Assess the Roles of Different Clustering Systems and Their Impact on Secondary Aerosol Formation^a^

simulation	clustering chemistry	reference/notes
*BaseCase*	SA-NH_3_*, SA-DMA*, IA-HIO_2_	(21,28,40)
*AN-AD*	SA-NH_3_*, SA-DMA*	(21,40)
*AN-AD-ID*	SA-NH_3_*, SA-DMA*, IA-DMA	(21,27,40)
IA-*scaling*^†^	SA-NH_3_*, SA-DMA*, IA-HIO_2_	(21,23,28,40)

a Star (*) indicates the inclusion of ionizing species for ion-mediated clustering, and cross (†) indicates simulation where the IA reactions by Finkenzeller et al., 2022, were not included. It should be noted that the simulated AN-AD-ID does not include IA-HIO_2_ clustering.

## 3 Results and Discussion

### 3.1 Particle Formation at Aboa and Neumayer

In the following discussion, nucleation mode refers to particles within sizes 5–25 nm for Aboa and 10–25 nm for Neumayer, Aitken mode indicates particles between sizes 25 and 100 nm, and accumulation mode encompasses all particles between 100 and 820/220 nm (Aboa DMPS extends to 820 nm and SMPS at Neumayer extends to 220 nm).

**Figure 1:**
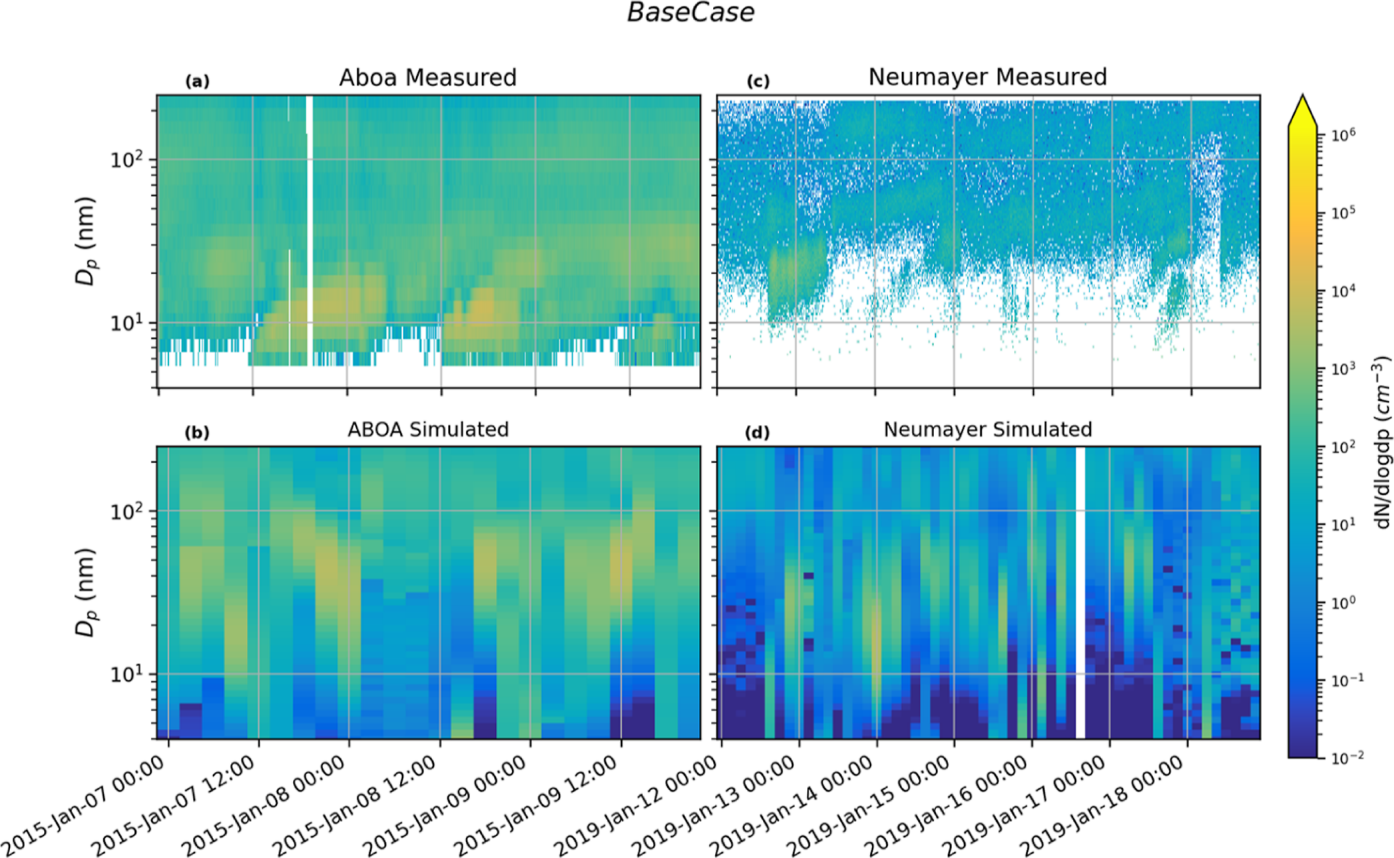
Measured particle number size distributions (d*N*/d log dp) at Aboa (a) and Neumayer (c) used for the *BaseCase* simulations. The modeled number size distributions are shown in panels (b,d).

The NPF and growth events observed at Aboa and Neumayer stations were compared to the simulated NPF and subsequent growth from the *BaseCase* setup in Figure 1. At Aboa, the simulation captures the NPF and growth events on the 7th of January, although the NPF event on 8th January is not captured (Figure S1, Supporting Information). The model overpredicts the total Aitken mode (25–100 nm) number concentrations (Figure S1 and Supporting Information Tables 1 and 2) at Aboa (measured relative Aitken concentration is 17.4% of the total number concentration, N*c*_tot_, while simulated Aitken mode number concentration is 75.8%, and mean Aitken number concentration Ait_mod_/Ait_meas_ ∼ 3.6), while underpredicting the nucleation mode contribution to N*c*_tot_ (15.4.% for simulation and 75.8% for observed, Nuc_mod_/Nuc_meas_ ∼ 0.18). On the other hand, the fractional contribution of accumulation mode number concentrations to the total number concentrations are similar for both simulation and measurement (6.7 and 12.4.%, Acc_mod_/Acc_meas_ ∼ 1.6). The underestimation of relative nucleation mode contribution to the N*c*_tot_ at Aboa in the simulations may arise from unaccounted NPF precursor emission sources (such as unregistered sea-bird colonies) in the model. On the other hand, the higher simulated relative Aitken mode number concentration indicates formation of new particles over the open ocean, and the ensuing growth of these freshly formed particles over land for a few hours before the air mass arrives at the measurement station.

At Neumayer, the model captures the particle formation event on the 12^th^ of January, but predicts a formation event on the 14th of January, which is not observed at the measurement station. The simulations accurately predict particle formation and inefficient growth on the 18th of January when compared to the NAIS measurements from Neumayer (see Figure S2, Supporting Information). The simulated contributions of both Aitken and accumulation mode particles to the N*c*_tot_ are in good agreement with the measured values. However, the simulations overpredict the mean Aitken and accumulation mode concentrations by a factor of ∼1.9 (Ait_mod_/Ait_meas_) and 1.23 (Acc_mod_/Acc_meas_), respectively (Table S2).

**Figure 2:**
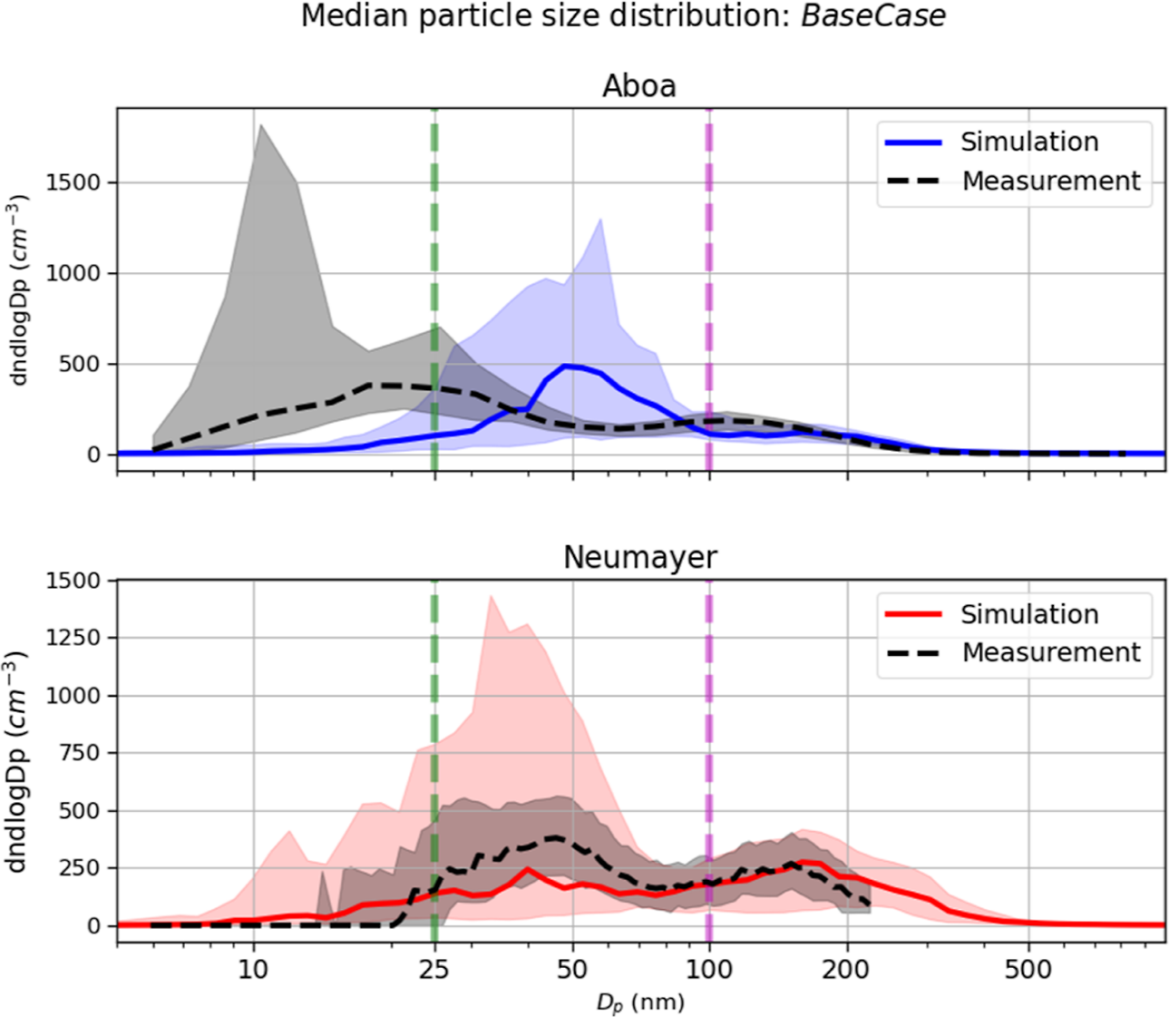
Median number size distributions at Aboa (upper panel) and Neumayer (lower panel) for the *BaseCase* simulations. The shaded areas indicate the 25th and 75th percentile range. The D_p_ between the green and magenta dashed line (25–100 nm) indicates the Aitken mode range, and the D_p_ above 100 nm (and <1 μm) represents the accumulation mode.

Figure 2 shows the simulated and measured median particle number size distributions at both Aboa and Neumayer. The simulated Aitken mode concentrations are overestimated at Aboa, with the peak Aitken mode diameter shifted to larger sizes (Figure 2, upper panel), while at Neumayer, the simulated median Aitken mode concentrations were underestimated in comparison to the measured median concentrations. The simulated median accumulation mode concentrations are underpredicted at Aboa, while at Neumayer, the simulated median accumulation mode concentrations agree well with the measurements. It should also be noted that at both sites, the Hoppel minima^41,42^ are well captured by the model, even though at Aboa, the modeled Hoppel minimum diameter is located at a larger size (∼100 nm), compared to the measurements (∼60 nm). Since the cloud supersaturations play a key role in the appearance of Hoppel minima in marine air masses,^41−43^ the location of simulated minima at larger diameters at Aboa could indicate that the cloud supersaturation along air masses arriving at Aboa is higher than the assumed cloud supersaturation in the model (*S*_c_ = 0.5%). There could be other reasons as to why the modeled Hoppel minima are at a larger diameter. It could be because the last aerosol cloud processing events in the model may generally have occurred too many hours upwind from the Aboa station or because the condensation growth after the last cloud passage is overestimated in the model. After a cloud passage, the particles will continue to grow and shift the Hoppel minimum toward larger sizes. At Neumayer, the modeled minima at ∼75 nm in diameter are in good agreement with the measurements (∼65 nm).

**Figure 3:**
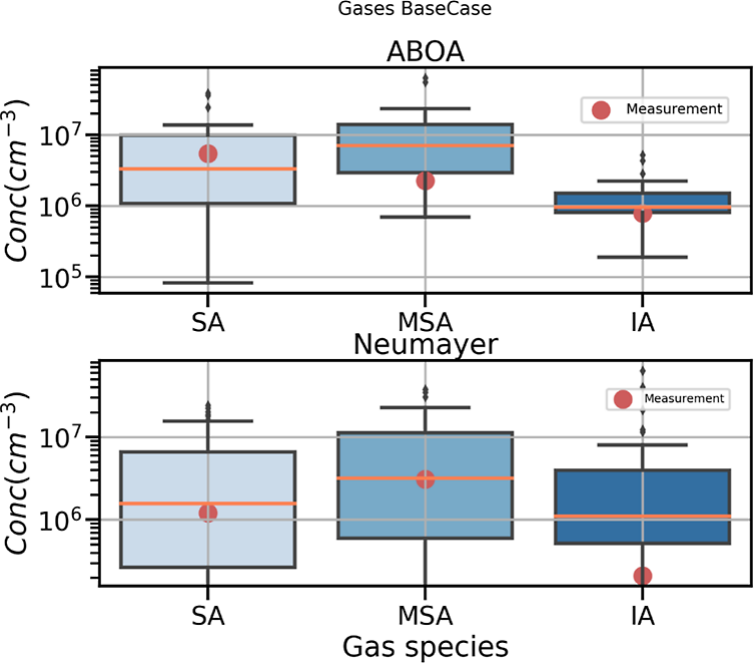
Gas-phase concentrations at Aboa (upper panel) and Neumayer (lower panel) for the *BaseCase* simulations. The red dots indicate the measured median values for the selected period. The central coral colored line in the box represents median values, while the whiskers indicate the maximum and minimum values. It should be noted, however, that the measurements at Neumayer are incomplete, with gaps in the data for the selected period.

The simulated and modeled gas-phase concentrations at both Aboa and Neumayer are shown in Figure 3. The modeled and measured median values for H_2_SO_4_ are in good agreement with both Aboa and Neumayer. Although the model tends to overestimate [MSA] at Aboa, the simulated values at Neumayer agree well with the measured [MSA] at the coastal site. The simulated and measured [IA] median values are in good agreement at Aboa, even though the model fails to capture the trend of measured [IA] at the site (Figures S3 and S4, Supporting Information). The measurements indicate a unique trend that the [IA] concentrations are highest during the early mornings (dawn) and evenings (dusk) and lowest during the noon, while the simulation indicates an opposite trend with [IA] peaking during the noon and decreasing at night. This peak in [IA] in the model is mainly due to the precursor species being photolyzed or reacting with OH to form [IA], which is efficient during the day. The measured trend of [IA] has also been observed in the SO Antarctic circumnavigation expeditions and is hypothesized to be related to either the efficient reaction of IA precursors by OH and/or HO_2_ or IA/IO itself being photolyzed during the day.^44^ However, the simulated trend of an increase in [IA] during daytime has earlier been observed in field campaigns conducted at the Maïdo observatory in the Reunion Island,^23,45^ SMEAR I,^46^ Michelstadt-Vielbrunn/Odenwald in Germany,^47^ at the Marambio station on the Antarctic peninsula,^13^ and in polluted urban cities of China (Beijing and Nanjing).^48^ Recent CLOUD chamber studies have shown that in typical Antarctic and SO environments where the median IA/H_2_SO_4_ ratios are 0.1 or higher, IA can increase particle formation rates by a factor of ∼10 or more compared to the H_2_SO_4_–NH_3_ pathway.^49^ This implies that even at noon when [IA] values are low in certain regions, it is sufficient to participate and drive nucleation. The *BaseCase* simulation included additional [IA] pathways based on the work by Finkenzeller et al., 2022 (reactions R1 and R2 in the Supporting Information). Figure S5 (Supporting Information) shows the gas-phase concentrations for the IA*-scaling* sensitivity simulations which indicates that omitting the reaction pathways suggested by Finkenzeller et al., 2022, results in underpredicting [IA], at both Aboa and Neumayer.

**Figure 4:**
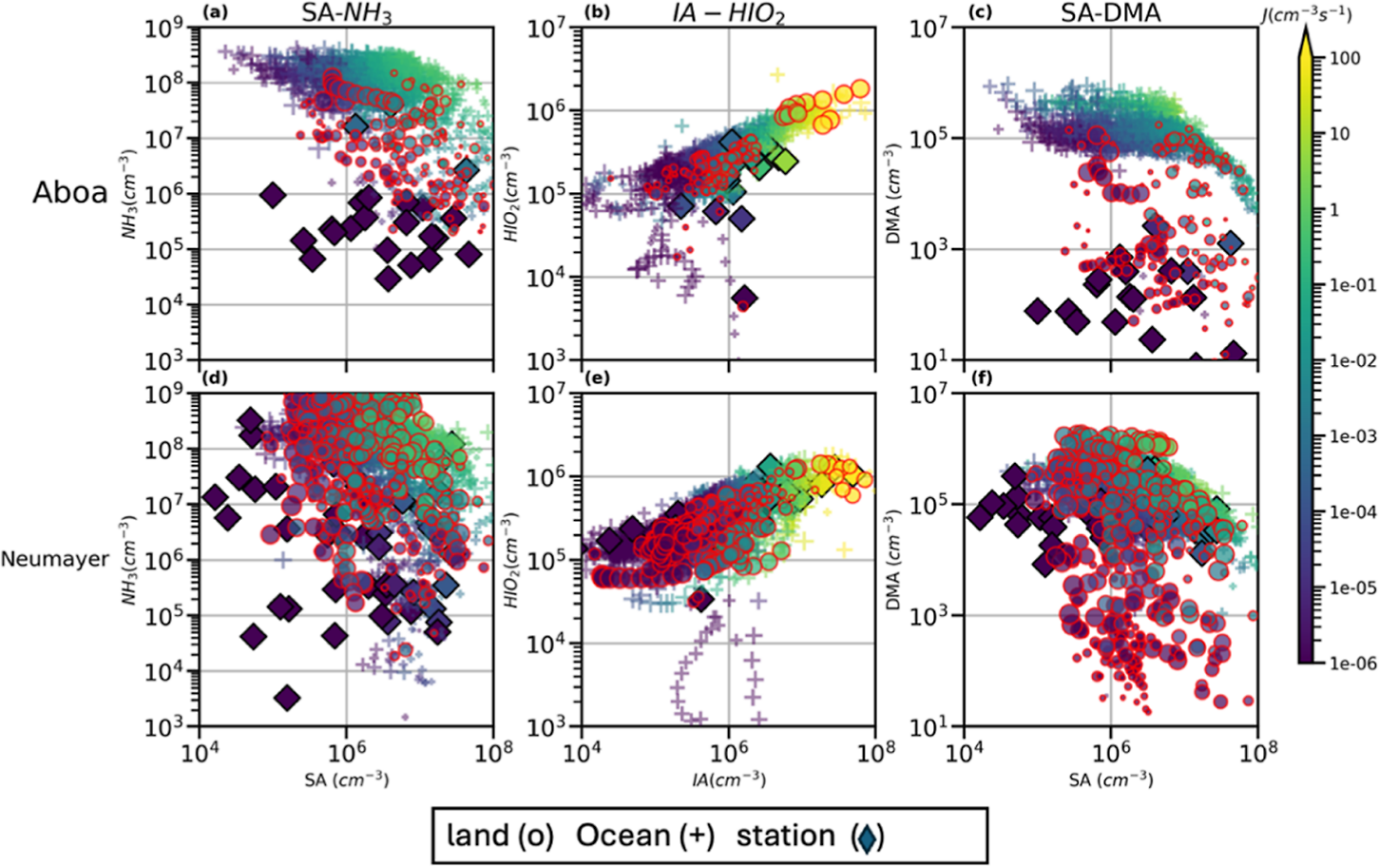
Simulated formation rates (*J*, cm^–3^) along the trajectory for all simulations and at the stations, Aboa and Neumayer (upper and lower panel), via the SA-NH_3_ (a,d), IA-HIO_2_ (b,e), and SA-DMA (c,f) and pathways for the *BaseCase* simulations. The “o” symbol with red edges indicates J values over land, “+” represents the values over ocean, and the diamonds represent the station values. The sizes of dots and stars indicate how far the air mass was from the station, with smaller points indicating air masses closer to the station and larger points indicating air masses further away from the station. Since the simulated J_SA-DMA_ values over land and sea for Neumayer (panel d–f) are similar in magnitude, the J values over sea are overlapped by the J values over land, i.e., the “+” are underneath the red “o”. Data points with formation rates of less than 10^–6^ cm^–3^ are excluded from the figure.

The simulated contribution of IA nucleation pathways to the formation of new particles is comparable to or in some cases even greater than the SA-NH_3_ pathway at both Aboa and Neumayer (Figure 4). At Aboa, the simulated IA-HIO_2_ formation rates are higher compared to the SA-NH_3_ formation rates, while at Neumayer, the simulated formation rates of IA-HIO_2_ are similar on a few occasions or higher than the SA-NH_3_ formation rates. This is consistent with experiments in the CLOUD chamber, which showed that at similar acid concentrations (SA vs IA between 10^6^–10^7^ cm^–3^), the IA-HIO_2_ system’s efficacy at forming new particles exceeds that of SA-NH_3_.^50^ Although HIO_2_ is an acid, it can play a key role in stabilizing neutral IA through strong binding and base-like behavior by proton transfers.^28,50^ It is plausible that the low simulated SA-NH_3_ formation rates at both Aboa and Neumayer are due to increased condensational scavenging of gas-phase SA by the IA-HIO_2_ particles (see Figure S9 and Table S3), thereby reducing the concentration of gas-phase SA available for clustering with either NH_3_ or DMA. Another reason for the low simulated SA-NH_3_ formation rates could be the cluster scavenging by larger particles, which are formed relatively more in the *BaseCase* than in *AN-AD* simulation. The gas-phase concentrations of HIO_2_ at Aboa are below the detection limit of CI-API-TOF.^9^ Even though the formation pathway for gas-phase HIO_2_ is uncertain in ambient conditions, the role of IA-assisted NPF cannot be ignored because IA can possibly cluster with other bases such as DMA and NH_3_. Observations at Aboa indicate SA-IA-NH_3_ clusters, albeit at lower concentrations, hinting at limited IA participation in the NPF to some extent.

The SA-DMA pathway on the other hand has a small influence on the NPF rates since it depends on the concentrations of the strong base DMA, whose concentrations are comparatively lower than NH_3_. Although the influence of SA-DMA on particle formation was small at Aboa and Neumayer, the simulated formation rates are higher over the ocean, which has also been observed onboard a ship measurement campaign and at the coastal station of Juan Carlos I (62.66° S, 60.39° W).^3^ Sensitivity simulations involving IA-DMA (*AN-AD-ID*) also show high J_IA-DMA_ (Supporting Information Figure S11), which is likely affected by the overprediction of formation rates due to the nature of the thermochemistry method (RI-CC2) used. Although, the formation rates for IA-assisted NPF are comparable to or higher than those of the SA-NH_3_ pathway, one must also note that there are possible uncertainties associated with missing DMA and NH_3_ sources (e.g., bird colonies, sea-flux emissions), which can affect the formation mechanisms dependent on these bases.

Figure 4 shows that the concentrations of SA and NH_3_/DMA are higher over the open sea and lower over land in air masses arriving at Aboa. In the Aboa simulations, both IA and HIO_2_ values are still high over the land since the precursor species CH_3_I has an approximate lifetime of ∼7 days.^51,52^ This pattern is especially evident at Aboa, a station that is ∼130 km inland, whereas at Neumayer, this distinction is not clear since the station is much closer to the coast. Simulations for Aboa show that J_SA-NH3_ is dominant over J_IA-HIO2_ on land, especially when the air mass is closer to the measurement station, while at Neumayer, although J_IA-HIO2_ is higher than J_SA-NH3_ on few occasions closer to station, the contribution of both these pathways is consistently similar (Figure S12, Supporting Information). The relatively higher J_IA-HIO2_ at Neumayer can be due to the air mass spending very less time over land as opposed to the sea (∼28% over land for Neumayer simulations Figure S13, Supporting Information).

The particle-phase concentration relative contributions in Figure S21 indicate that sulfate [S(VI), sulfur with oxidation number 6] dominates the nucleation mode (1–25 nm) mass at both Aboa and Neumayer with ∼55 and ∼52% mass fractions, respectively, while NH_4_^+^ contributes ∼10% at both stations to the particle mass fraction (values in Figure S14, Supporting Information). At Aboa, the average particulate ammonia contribution was between 1 and 7% of the cluster mass (cluster with at least 4 SA molecules).^9^ The simulations agree well with the measurements, wherein for particles <3 nm, the ammonium mass contribution is ∼10% for the entire simulated period. Aqueous-phase formation of MSA governs the growth of particles in the Aitken mode (25–100 nm, ∼ 44 and 40% at Aboa and Neumayer, respectively), while the sea-salt constituents of Na^+^ and Cl^–^ contribute more to the accumulation (100 nm −1 μm) and coarse (1–10 μm) mode particle mass. Although S(VI), MSA, and NH_4_^+^ are the major contributors to the particle mass in nucleation mode, IO_3_^–^ also plays a small but significant role in contribution to mass in the nucleation to accumulation mode particles. It should also be noted that the IO_3_^–^ contribution is greater at Neumayer compared to Aboa, and this is mainly attributed to the coastal location of Neumayer (IA is formed over open oceans in the simulations). Although IA can substantially assist particle formation over the oceans, it is not an efficient driver of particle growth, which is dominated by S(VI), MSA, and NH_4_^+^. At both Aboa and Neumayer, MSA participates in the growth of particles >10 nm via heterogeneous formation in the aqueous phase, which agrees well with observations made onboard icebreaker campaigns in the SO.^44^

### 3.2 Role of Iodine-Driven NPF

**Figure 5:**
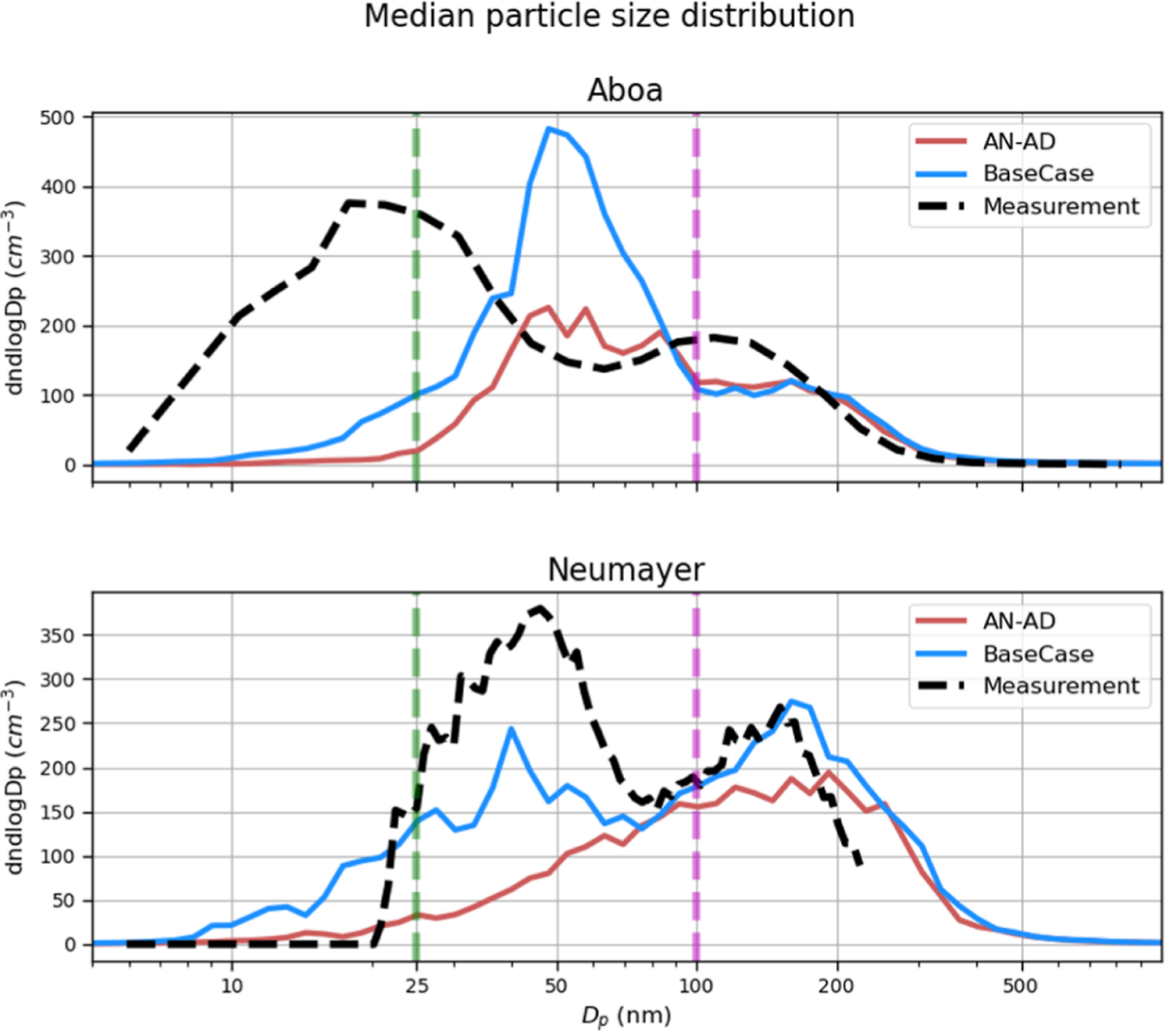
Median size distributions at Aboa (upper panel) and Neumayer (lower panel) for the *BaseCase* and *AN-AD* simulations. The D_p_ between the green and magenta dashed line (25–100 nm) indicates the Aitken mode range, and the D_p_ above 100 nm (<1 μm) represents the accumulation mode.

The simulated role of IA-driven pathways in the formation and growth of particles in the marine boundary layer in the simulations is evident in the median particle number size distributions for different sensitivity tests, as shown in Figure 5. *AN-AD* simulations, which do not take IA-base nucleation into account, lack a clear Aitken mode at Neumayer implying that SA-NH3 and SA-DMA particles formed over the SO have already grown to larger sizes or have been lost by coagulation to larger sized particles. When IA-HIO_2_ particle formation is considered, more particles are formed along the air masses which creates an Aitken mode that is missing in the *AN-AD* simulations, improving the comparison to the measurements. This also holds true for simulations involving the clustering of IA-DMA (Figure S15, Supporting Information), which also shows an even better representation of Aitken mode particles than the simulations with IA-HIO_2_. Based on the above argument, we concur that including iodine-driven particle formation pathways alongside SA-NH_3_ improves the particle number size distribution. But where does IA-driven particle formation play a role if not at the station?

Earlier observations at Aboa indicated that the NPF events observed at the station were predominantly via the ion-mediated SA-NH_3_ pathway with no pure IA clusters (IA-base) observed.^9^ However, iodine clusters (SA-IA-NH_3_ trimers) were observed at lower concentrations, indicating that iodine-enhanced pathways can potentially participate in particle formation. However, the lack of freshly formed particles via the SA-NH_3_ pathway at Aboa in our simulations indicates incomplete information on the various sources of bases in the model, especially over land which can participate in NPF. Considering that the air mass traverses over the open SO for long periods (average time spent over sea was > ∼88% for air masses arriving at both stations, Supporting Information Figure S18 ), it is plausible that iodine-driven NPF indeed plays a crucial role over these open waters. In typical SO environments with low median IA/H_2_SO_4_ ratios of ∼0.1, IA can enhance formation rates (J_HIO*x*–H2SO4–NH3_) by a factor of ∼10.^49^ However, at inland sites such as Aboa for the same enhancement effect of ∼10, the required HIO_3_/H_2_SO_4_ ratio is ∼0.8.^49^ Accounting for a potential synergistic HIO_*x*_–H_2_SO_4_–NH_3_ pathway, we speculate that this pathway can increase the NPF and Aitken mode number concentrations over SO, where the simulated [NH_3_] concentrations are a few ppt. However, over land and close to Aboa measurement where the simulated mean [NH_3_] is ∼0.1 ppt, the formation rates would be ∼10 times lower and closer to the simulated J_HIO3–HIO2_, between 0.1 and 10 s^–1^, suggesting that local missing NH_3_ emissions could be the limiting factor in simulating sub-10 nm particle formation. Since the formation rates of IA-HIO_2_ clustering mechanism are similar to or exceed the SA-NH_3_ rates over the ocean (Figure 4), it is possible that these IA-HIO_2_ clusters constitute additional sites (in addition to SA-NH_3_ or SA-DMA clusters), onto which inorganics such as SA condense. The subsequent growth of these particles is facilitated by heterogeneous reactions (for e.g., the aqueous-phase production of MSA) resulting in growth to larger Aitken mode sizes (Figures S16,S17). Conversely, a lack of IA-base clusters would result in fewer new particles over the ocean, which explains the lack of a prominent Aitken mode seen in the simulated results.

The simulated mean particle-phase compositions (normalized mass fractions) along the trajectories show that in the sub-10 nm size range, the IO_3_^–^ contribution is slightly higher in air masses traversing over the open sea for the Aboa case which is located inland (Figures S16 and S17, Supporting Information); for the coastal site Neumayer, the fractions are similar. The contribution of IA to particle mass (∼<1% at Aboa and ∼4% at Neumayer) over land is minor compared to the S(VI) (∼66 and 67%) at both measurement stations, indicating that the growth of particles in this size range is dominated by sulfur species. Since IA-assisted particle formation is important over the open ocean compared to over land, it is plausible that the increase in smaller particles (via the IA-base pathway in addition to the SA-driven pathway over the sea) increases the likelihood of more particles growing to Aitken mode sizes via further condensation of SA and heterogeneous reactions (Figure S18, Supporting Information).

Regardless of the role of different precursors in particle formation, the uncertainties associated with quantifying the role of these precursors should be highlighted. Apart from the known measurement uncertainties (cf. Supporting Information), we would like to highlight that the formation rates of the IA-HIO_2_ system were found to be sensitive to the gas-phase concentrations of HIO_2_ (Figure S8 and Reaction R3, Supporting Information). The gas-phase formation mechanism of HIO_2_ is unknown, which together with the fact that the diurnal profile of IA is not well understood adds further restraint on the quantification of the IA-HIO_2_ system to particle formation. Additionally, since the NPF mechanisms are complex and rather diverse, the possibility of IA/HIO_2_ participating in the stabilization of other acids/bases chemistries cannot be ruled out (SA-IA-NH_3_ was observed at Aboa).

Table S4 shows the impact of iodine-assisted particle formation on CCN number concentrations. According to the model, iodine-assisted particle formation increases the CCN number concentrations at both Aboa and Neumayer by ∼101 and ∼21%, respectively, at *w* = 0.6 ms^–1^. On the other hand, at *w* = 0.1 m s^–1^, iodine decreases CCN concentrations at Aboa by ∼5%. This is due to the rapid adiabatic cooling at higher updraft velocities which result in higher cloud water-vapor saturations, thereby allowing smaller particles to be activated to CCN. Since at *w =* 0.1 ms^–1^, the median S_c_ = ∼0.15%, fewer particles in the *BaseCase* simulation get activated to CCN when compared to the *AN-AD* simulation (median activation diameter, Dp_act_, for both cases ∼78 and 80 nm, respectively, Figure S20, Supporting Information). From Figure 5, we can see that at Aboa, the *AN-AD* simulation exhibits particle concentrations higher than those of *BaseCase* in the Dp_act_ range of ∼78–80 nm. A plausible reason is the availability of more Aitken mode particles in *BaseCase*, onto which the gaseous precursors can condense, rather than larger particles, thereby reducing the number of particles close to Dp_act_. The relative changes to CCN number concentrations are important since they can impact the radiative balance, especially in the SO.^53,54^

In conclusion, our results demonstrate the complexity of aerosol processes occurring in pristine environments, such as Antarctica. Our findings suggest that only considering SA-driven nucleation along the air mass trajectories may be insufficient in explaining the measured Aitken mode particle number concentrations at both measurement stations. Incorporating both iodine-driven (IA-HIO_2_/DMA) and SA-driven particle formation mechanisms improves the modeled particle number size distribution representation. Iodine-assisted particle formation is significant over the open ocean, where it can provide additional sites which subsequently grow by condensation of gaseous species and heterogeneous reactions. Even though iodine-assisted particle formation can improve the comparison of modeled and observed Aitken mode particle number concentrations, we are still unable to simulate the local secondary aerosol formation, which is responsible for the observed particles in size ranges <10 nm at Aboa. This is most likely because of missing information on sources of various bases (e.g., NH_3_) over land that participates in NPF. It is plausible that potential overestimation of simulated particle growth rates can also lead to an overestimation of Aitken mode particle number concentrations and a subsequent underestimation of nucleation mode number concentrations. The impact of iodine-assisted particle formation on CCN concentrations is large for moderate to high updraft velocities (*w* > 0.5 m s^–1^) at Aboa, while at Neumayer, the CCN concentration changes were modest. These results indicate that accounting for the contribution of iodine species to aerosol formation (e.g., IA various bases, SA-IA, and SA-IA-NH_3_) may provide an improved representation of natural aerosol sources and the related forcings in a pristine marine environment. This is essential for assessing the effects of anthropogenic aerosol sources on the overall forcing at present and in future, which is important in Antarctica and other polar regions due to possible impacts on temperature and sea ice extent. Such natural aerosol sources can also contribute to feedback processes, like increased negative forcing due to more natural aerosol formation resulting from decreasing sea ice. Assessing the potential role of iodine species in such feedback loops by means of adequate representation is an essential building block in capturing such patterns in climate projections. The importance of iodine species in marine and coastal NPF and the role of HIO_2_ as a stabilizer have been experimentally verified in laboratory conditions, and in our study, we apply the best available molecular thermodynamic data for modeling the neutral iodine oxoacid and iodine-base cluster formation. However, an essential factor affecting the model predictions is the uncertainties associated with precursor emissions and gas-phase chemistry, e.g., the HIO_2_ formation pathways. Although the median [IA] at Aboa agrees with the measured concentrations, further investigation in understanding the diurnal profile of IA at certain locations should be prioritized. The improved understanding of gas-phase chemistry, for e.g., photolabile precursor production/loss reactions, will play a vital role in limiting the uncertainties in NPF pathways and contribute to ascertaining the role of iodine species to particle formation and growth to CCN-relevant sizes in such pristine regions.

## Supporting Information Available

Description of the model framework detailing the FLEXPART simulations, emission data used as input to ADCHEM, and molecular thermochemistry data used for simulating particle formation in the ClusterIn plugin; details about the measurement site and the instrumentation used during the Aboa and Neumayer II campaigns; total number concentration model-measurement comparison in the nucleation; Aitken and accumulation mode at both stations; relative contributions of the different particle sizes to the total particle concentrations; ratio or mean simulated to measured number concentrations in each of the modes; NAIS measurement comparison with simulated particle size distribution; model-measurement comparison of SA, IA, and MSA time series; mean diurnal profiles for the BaseCase simulation; box plots depicting the model-measurement comparison of gas-phase SA, IA, and MSA for IA-scaling simulation; modeled iodic acid and iodous acid production for conditions resembling a molecular iodine photolysis experiment in the CLOUD chamber; comparison of simulated iodous acid concentrations for the IA-scaling and BaseCase; IA formation reactions; comparison of SA gas-phase concentrations in air masses spending time over land and sea for both Aboa and Neumayer; measured and simulated median SA gas-phase concentrations at the respective stations; simulated formation rates (*J*_*SA-NH3*_ and *J*_*SA-DMA*_) at the stations for the BaseCase and AN-AD and for AN-AD-ID case; ratio of J_SA-NH3_/J_IA-HIO2_ for air masses over land and sea arriving at both Aboa and Neumayer stations; fraction of time the air mass has spent over land and sea; normalized contribution of different species (S(VI), Cl^–^, Na^+^, NH_4_^+^, MSA, and IO_3_^–^) in distinct size modes at Aboa and Neumayer; comparison of median PSD for the BaseCase, AN-AD, AN-AD-ID, and measured PSD; normalized particle phase mass for air masses traversing over land and sea in each size mode arriving at Aboa and Neumayer; median PSD in land and sea air masses arriving at both stations; mean trajectories arriving at Neumayer and Aboa; simulated activation diameter (Dp_act_); particle-phase relative fractions for different species at the two stations; and median change in CCN number concentration (ΔCCN) due to the iodine-assisted particle formation, relative to *AN-AD* simulation for 3 updraft velocities (*w* = 0.1, 0.6, and 1.0 m s^–1^) (PDF)
