# Supplementary material for: Role of Iodine-Assisted Aerosol Particle Formation in Antarctica
Source: Environ Sci Technol. 2024 Apr 16;58(17):7314–24. doi: 10.1021/acs.est.3c09103 (PMC11064213; doi:10.1021/acs.est.3c09103)
Supplement: Supplementary file 1 — es3c09103_si_001.pdf [file es3c09103_si_001.pdf]

Supporting information for: Role of iodine-assisted aerosol particle formation in Antarctica

Carlton Xavier*, Robin Wollesen de Jonge, Tuija Jokinen, Lisa Beck, Mikko Sipilä, Tinja Olenius, Pontus Roldin

Department of Physics, Lund University, Professorsgatan 1, Lund, SE-22363, Sweden

Swedish Meteorological and Hydrological Institute (SMHI), Norrköping, SE-60176

Email: carlton.xavier@fysik.lu.se

Phone: +46709711796

This PDF includes:

Model framework- S2
Measurement site and instrumentation- S3
Figure S1, Table S1, S2- S4
Figure S2- S5
Figure S3- S6
Figure S4- S7
Figure S5- S8
Figure S6- S9
Figure S7- S10
Figure S8- S11
Figure S9, Table S3- S12
Figure S10- S13
Figure S11- S13
Figure S12- S14
Figure S13- S15
Figure S14- S15
Figure S15- S17
Figure S16- S18
Figure S17- S19
Figure S18- S20
Figure S19- S21
Figure S20- S21
Figure S21, Table S4- S22

Model framework

The Aerosol Dynamics, gas and particle phase CHEMistry and radiative transfer model (ADCHEM)[1,2], a 1-d column model, using 40 vertical layers (logarithmically spaced) extending up to ~2600 m was run as a Lagrangian model along air mass trajectories arriving at the measurement station (Aboa and Neumayer) every 3rd hour. ADCHEM model time step for aerosol dynamics and gas-particle chemistry is 30 seconds. The air mass trajectories and potential emission sensitivity fields were generated using FLEXPART v10.4[3,4.] A 7 day backwards in time air mass history for all trajectories arriving at the measurement stations (every 3rd hour i.e., 8 trajectories per day) were used in these simulations. ECMWF ERA5 reanalysis meteorological data (0.5^o x 0.5^o and 137 height levels and 1-hour temporal resolution) was used as an input to FLEXPART. FLEXPART derived normalized potential emission sensitivity fields were combined with oceanic emissions and anthropogenic emissions from global inventories.

Emissions of marine gas-phase species such as iodomethane (CH_3I), tribromomethane ($CHBr_3$) and DMS were retrieved from CAMS-OCE and global oceanic emissions (CAMS-GLOB-OCE, v3.1 daily profiles 0.5^o x 0.5^o spatial resolution and daily means)[5,6]. Sea-surface temperature (SST) and wind speed dependent primary sea-spray aerosol emissions (valid in size ranges 10nm-10μm) were derived based on parameterizations outlined in Sofiev et al., 2011. The SSA parameterizations of Sofiev et al., 2011 shows an increase in emission flux for larger particles with increasing temperatures, while the emission flux of smaller particles decreases[7,8]. NH_3 emissions from seabird colonies were obtained from a global inventory[9]. Additional fluxes of NH_3 and DMA to/from the open oceans based on surface ocean equilibrium concentrations of NH_3 and DMA were estimated[10]. In this method pK_a for NH_3-NH_4^+ equilibrium is calculated based on SST and salinity.

ClusterIn uses most recent available data sets for molecular cluster thermochemistry, using combinations of SA, NH_3, DMA, IA and HIO_2. In these simulations we specifically, applied ion-mediated SA-NH_3 [11] and SA-DMA[12], pathways (ionization rate of 2 $cm^{-3}s^{-1}$) and neutral HIO_3-HIO_2 [13] (IA-HIO_2), and HIO_3-DMA [14](IA-DMA). For more details about the quantum chemical level of theory used in the datasets please refer to the supplement section (Model framework). Here ion-mediated refers to chemistries which include both electrically neutral and charged clusters and involve the ionization and recombination through collisions with charger ions[15,16]. This implies that NPF includes contributions from both neutral and charged clusters, and pathways with different charging states interact with each other. The SA-NH_3 and SA-DMA data sets are both computed using the DLPNO-CCSD(T)/aug-cc-pVTZ//ωB97X-D/6-31++G(d, p) quantum chemical level of theory, while the IA-HIO_2 data set applies the DLPNO-CCSD(T)/M06-2X7/def2-TZVp level of theory. The IA-DMA is computed using the RI-CC2/aug-cc-pVTZ-PP//ωB97XD/6-311++G(3df,3pd) with aug-cc-pVTZ-PP basis set for iodine. Steady-state particle formation rates simulated using the thermochemical datasets for SA-NH_3 and IA-HIO_2 have previously been compared to experimental data from the CLOUDchamber and have agreed well with measurements performed at low temperatures and moderate-to-high base concentrations[11,13]. This good agreement with CLOUD

measurements can be expected to be due to the application of the highly accurate DLPNO method for single-point energy calculations, and the quasi-harmonic corrections employed in the calculations which generally reduce cluster over-binding[11]. Formation rates based on the SA-DMA quantum chemical data set have been shown to agree with the CLOUD experiments at both low and high DMA gas concentrations[17].

Measurement site and instrumentation

Particle number concentration and gas phase species data from two measurement stations were compared against model results. Both the Neumayer II (70.66° S, 8.27° W) and Aboa (73° 03'S, 13° 41'W) stations are located on Queen Maud land, Antarctica (Figure S19, supplement shows the 7-day air mass trajectories). During the measurement campaigns, the concentration of neutral SA, MSA and IA was measured with a chemical ionization atmospheric pressure interface time-of-flight mass spectrometer (CI-APi-TOF, Tofwerk AG & Aerodyne Research Inc.,[18]). The gas-phase measurements at Neumayer are incomplete, with gaps in the data for the selected period. The reagent ion used with the CI-APi-TOF was nitrate ion (NO_3^-), which enables the detection of low-volatility vapors. The CI-APi-TOFs were both calibrated using SA and thus, IA and MSA concentrations from the campaigns should be treated as low-level values (assuming kinetic limit charging).

For the measurement of ions in the size range of 0.8 - 40 nm and small particles from 2 - 40 nm, a neutral cluster and air ion spectrometer (NAIS, Airel Ltd.,[19]) was deployed in both campaigns. For the detection of particles a scanning mobility particle sizer (SMPS, 10-220nm, [20]) was used at Neumayer III and a differential mobility particle sizer (DMPS, 6-820 nm) at Aboa. At Neumayer III, the instrumentation was set up in two containers, about 1.5 km south of the main station. The SMPS was located in the Air Chemistry Observatory and the NAIS and CI-APi-TOF were temporarily placed in a container close to the Air Chemistry Observatory. The measurements took place from 17 November 2018 until 7 February 2019, but only the period 12-19th of January was selected for this analysis. This period was selected since it represents a whole week with both NPF and non-NPF event days. At Aboa, all instruments were placed in a container located about 200 m upwind of the station to prevent pollution from the station. The dates of measurements selected for this study are between 7 and 9 of January 2015. For a detailed description of the campaign in Aboa, we refer to Jokinen et al., 2018.

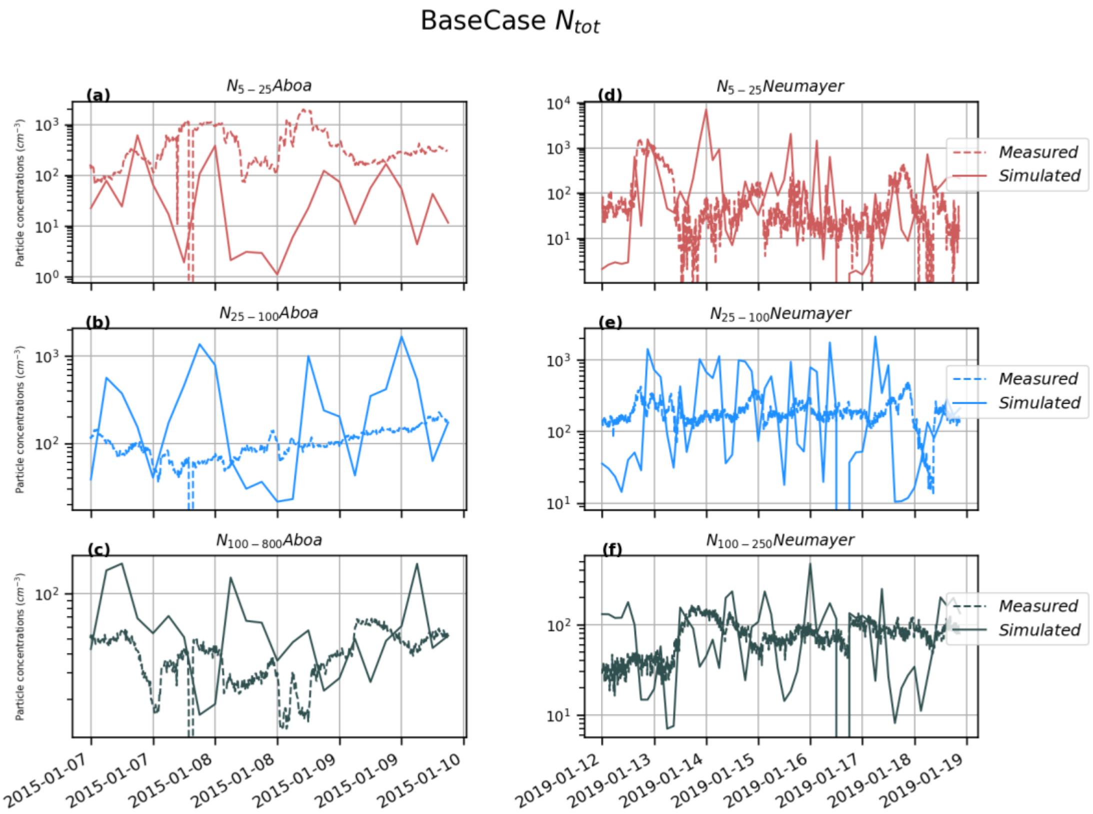

Figure S1. The total number concentrations (Nc_{tot}) of nucleation mode (5-25 nm for Aboa and 10-25 nm for Neumayer), Aitken mode (25-100 nm) and accumulation mode (100 – 840/220 nm) particles at Aboa (a,b and c) and Neumayer (d, e and f).

Table S1. Relative contribution of different particle sizes to the total particle number concentration (Nc_{tot}) for the *BaseCase* setup.

**Station**		**Nucleation mode (%)**	**Aitken mode (%)**	**Accumulation mode (%)**
**Aboa**	**Measured**	75.8	17.4	6.7
	**Simulated**	15.4	72.1	12.4
**Neumayer**	**Measured**	32.1	48.2	19.7
	**Simulated**	41.3	46.3	12.3

Table S2. Ratio of mean simulated number concentrations to mean measured number concentrations in each mode for the *BaseCase* setup.

	$\mathbf{Nuc_{mod}/Nuc_{meas}}$	$\mathbf{Ait_{mod}/Ait_{meas}}$	$\mathbf{Acc_{mod}/Acc_{meas}}$
**Aboa**	0.18	3.60	1.6
**Neumayer**	N/A due to very few or no measured	1.90	1.23

	particles below 20 nm		

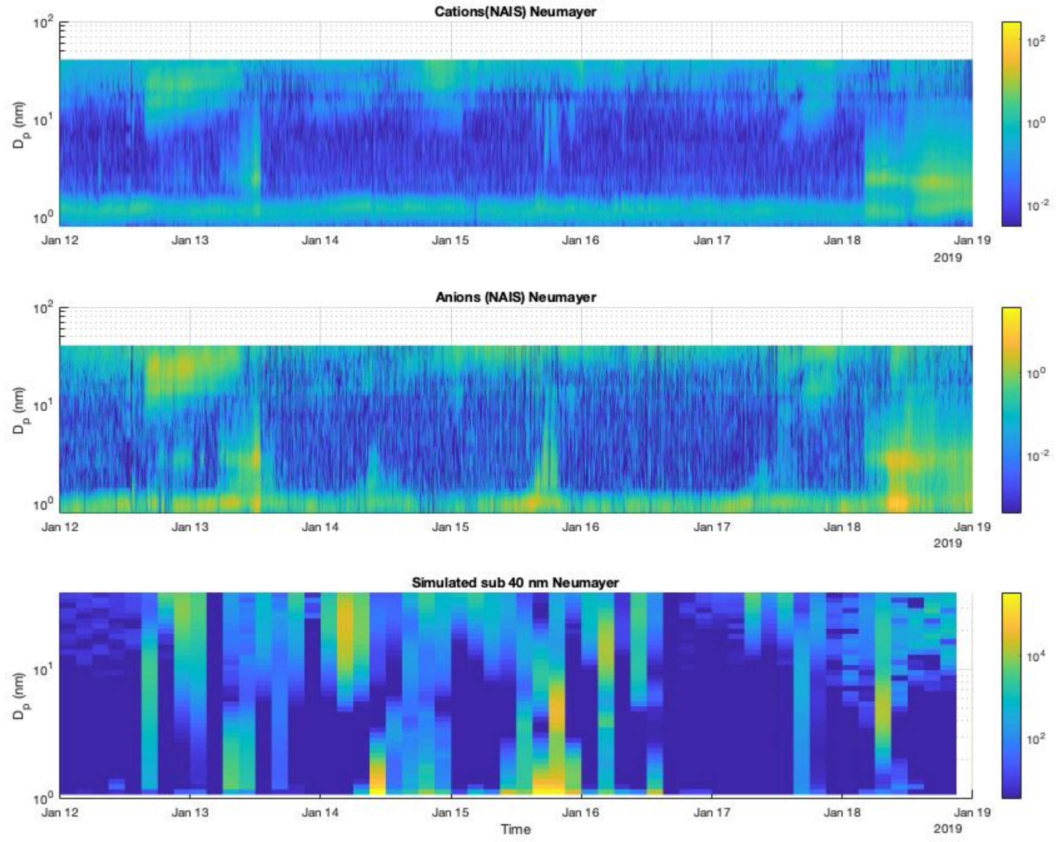

Figure S2. Cations (upper panel) and anions ions (middle panel) measured by NAIS, and the simulated particle size distribution (lower panel) at Neumayer.

The particle formation event and inefficient growth to Aitken mode on the 18th at Neumayer is well simulated by the model. The model also captures the formation events on January 13th and 16th well.

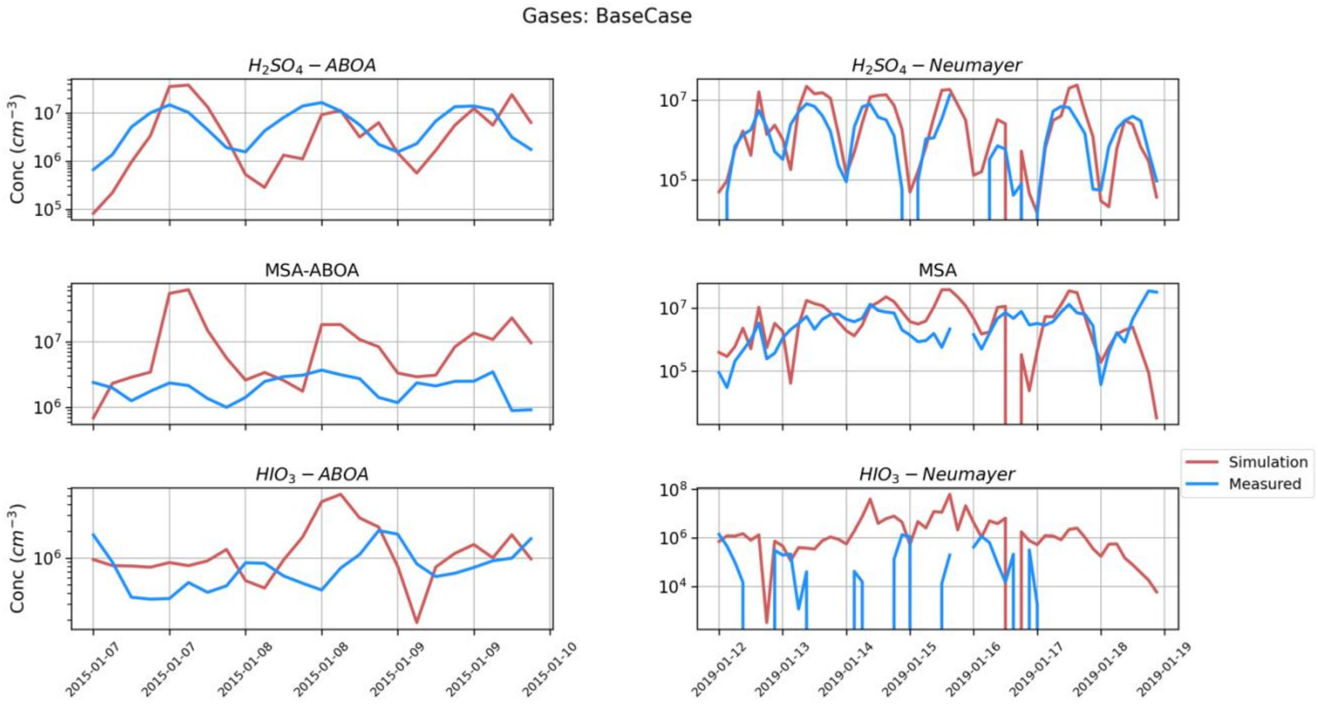

Figure S3. Gas phase concentration time series for the *BaseCase*. The left panels depict modeled and measured gas-phase concentrations of H_2SO_4, MSA and HIO_3 at Aboa, while the right panels show comparison for Neumayer.

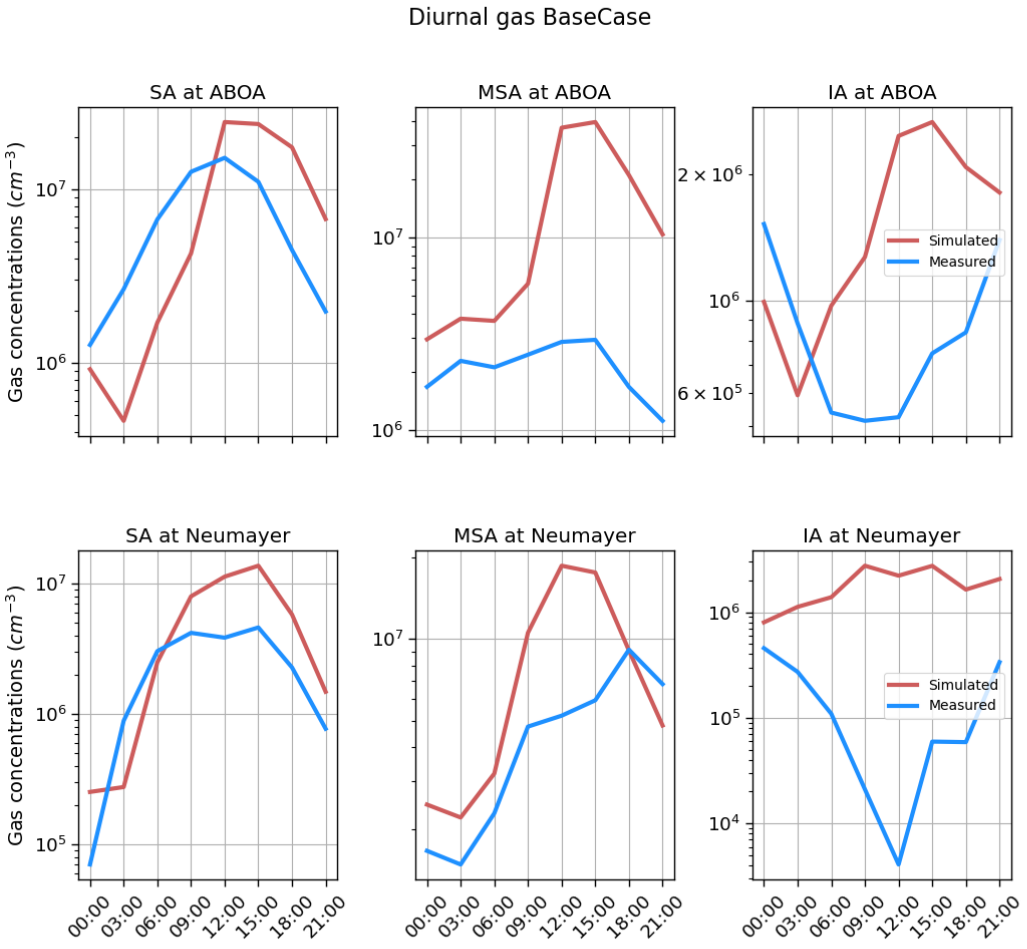

Figure S4. Diurnal mean gas phase concentrations for the *BaseCase* simulation. The upper and lower panels depict modeled and measured gas-phase concentration of H_2SO_4, MSA and HIO_3 at Aboa and Neumayer respectively.

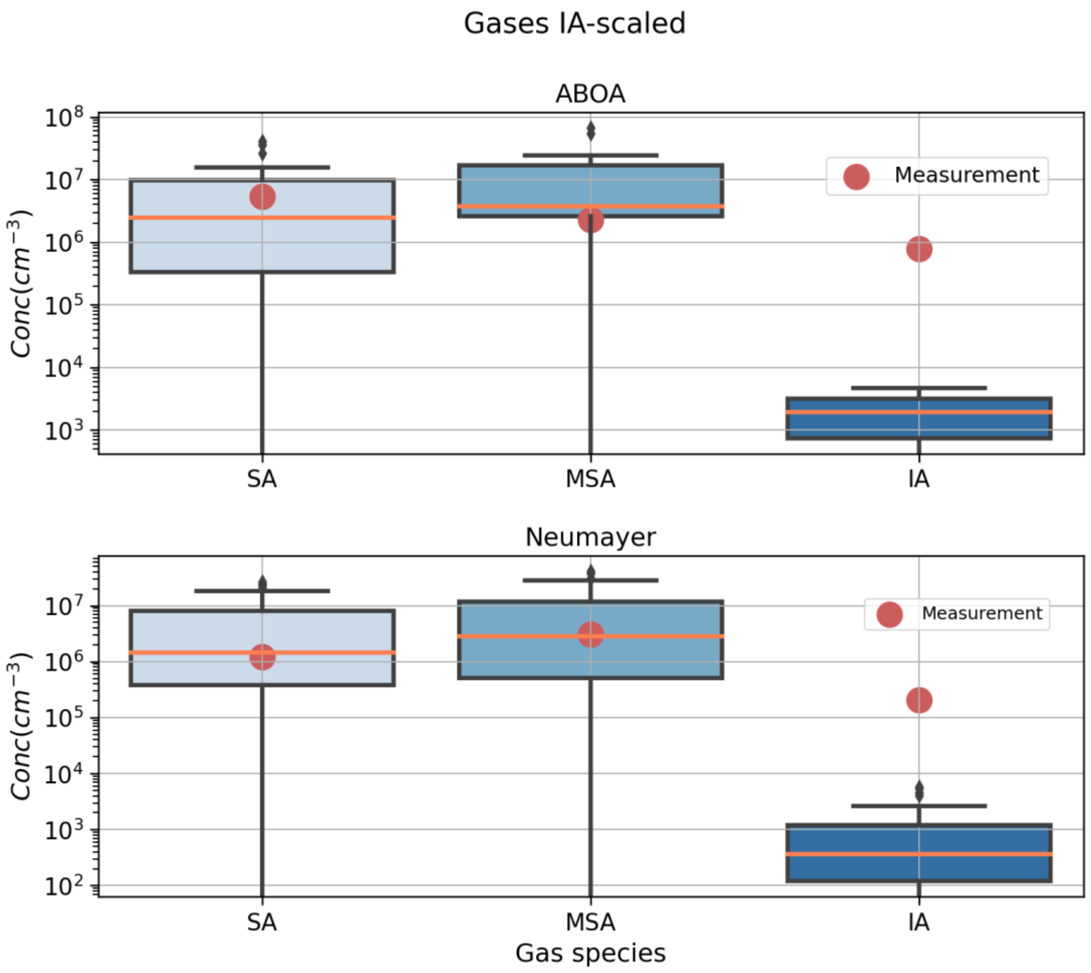

Figure S5. Gas-phase precursor concentrations at Aboa (upper panel) and Neumayer (lower panel) for the *IA-scaling* simulations. The red dots indicate the measured median values.

In the *IA-scaling* simulations the reactions for $HIO_{3(g)}$ production from Finkenzeller. et al[21], where not included. This resulted in lower gas-phase HIO_3 production compared to the *BaseCase* simulations. These sensitivity simulations were performed to indicate the improved model performance when these reactions are included. The two reactions omitted in *IA-scaling* simulations (based on Finkenzeller. *et al* [21]) were:

(R1) IOIO + O_3 → $IOIO_4$, k_1=1.5x10^{-13} cm^3 $molec^{-1}$ s^{-1}
(R2) $IOIO_4$ + H_2O → HIO_3 + HIO + O_2, k_2=2x10^{-16} cm^3 $molec^{-1}$ s^{-1}

The $HIO_{2(g)}$ pathway is largely unknown. In the model HIO_2 is formed by the following reaction.

(R3) IOIO + H_2O → HIO_2 + HOI, k_3=10^{-19} cm^3 $molec^{-1}$ s^{-1}

R3 was suggested as a possible reaction forming HIO_2 in the CLOUD experiments where I_2 was photolyzed by green light[3]. The reaction rate constant (k_3) was estimated by running

the aerosol dynamics and gas- and particle-phase chemistry kinetic multilayer model ADCHAM[22] for conditions resembling the CLOUD I_2 photolysis experiments by He *et al.*[3]. We applied a CLOUD chamber wall loss rate of 2.2×10^{-3} s^{-1} for HIO_3 and HIO_2 that are identical to the wall loss rate measured for H_2SO_4 in the CLOUD chamber[23]. In the model simulations we did not consider any particle formation. Thus, apart from the gas-phase chemistry degradation, HIO_3 and HIO_2 were only lost to the chamber walls in the model.

Figure S6 shows the modelled HIO_3 and HIO_2 concentrations for a CLOUD chamber experiment with fixed steady-state concentration of I_2 and ozone equal to 7 ppt_v and 40 ppb_v respectively. The RH was 69 % and the temperature -10 °C. The experiments (model simulations) were initiated without any light (dark chamber). A green light source was switched on to photolyze molecular iodine (I_2), which resulted in an I atom production rate of 6.2×10^4 molecules $cm^{-3}s^{-1}$. The model results are comparable with the observations presented in the Supporting Online Material Fig. S1 by He *et al*.[3]. According to the experiments the iodic acid concentration reaches a maximum concentration of ~8×10^6 molecules/cm^3 approximately 10 minutes after the green light is turned on in the chamber. The observed HIO_3 concentration then start to decrease slowly during the experiments and reach ~3×10^6 molecules/cm^3 >2 hours after the light was turned on. The gradual decrease in the observed HIO_3 can possibly be attributed to an increasing particle-phase condensation sink. Since the model did not consider any losses toward the aerosol particle-phase the HIO_3 concentration stabilizes at a fixed stead-state concentration of ~9×10^6 molecules/cm^3 within ~10 minutes. The observed HIO_2 concentrations stabilizes at a steady-state concentration of ~1.5×10^5 molecules/cm^3 while our modelled HIO_2 concentrations reach a steady-state concentration of ~1.8×10^5 molecules/cm^3.

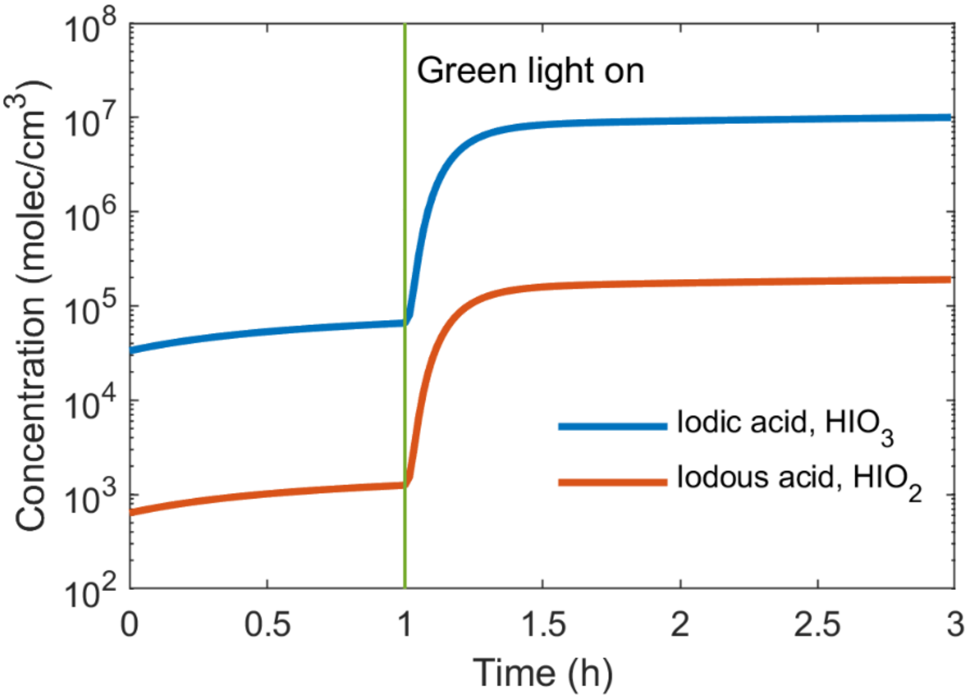

Figure S6. Modelled iodic acid and iodous acid production for conditions resembling a molecular iodine photolysis experiment in the CLOUD chamber. The experiment is described by He *et al.*[24] (see supplementary figure S1 in He *et al.*[24]).

Figure S7 shows the modelled concentrations of HIO_3 and HIO_2 for conditions resembling experiments in CLOUD with I_2 concentrations varying between 0.4 and 4 ppt_v, RH=40 % (34-44 % in the experiments) and $[O_3]$ =40 ppb_v (38-42 ppb_v in the experiments). The model results are comparable with the observations presented in the Supporting Online Material Fig. S2 by He *et al.*[24]. He *et al.* showed that the HIO_3 concentration increases approximately linearly with the I_2 concentration (I atom formation rate) while HIO_2 increases approximately with the square root of the I_2 concentration. The modelled HIO_3 trend agrees well with the observed trends in the CLOUD chamber, with modelled HIO_3 concentrations within a factor of 1.05-1.3 from the observations. However, in contrast to the observations in the CLOUD chamber, the modeled HIO_2 concentration also increases linearly with the I_2 concentration. This indicates that HIO_2 likely are formed by other reaction pathways than what was considered in this work. Still, the modelled HIO_2 concentration is within a factor of 0.5-2.0 from the observed HIO_2 concentrations in the CLOUD chamber, which we consider acceptable in this pioneering modelling study on iodine-assisted aerosol particle formation.

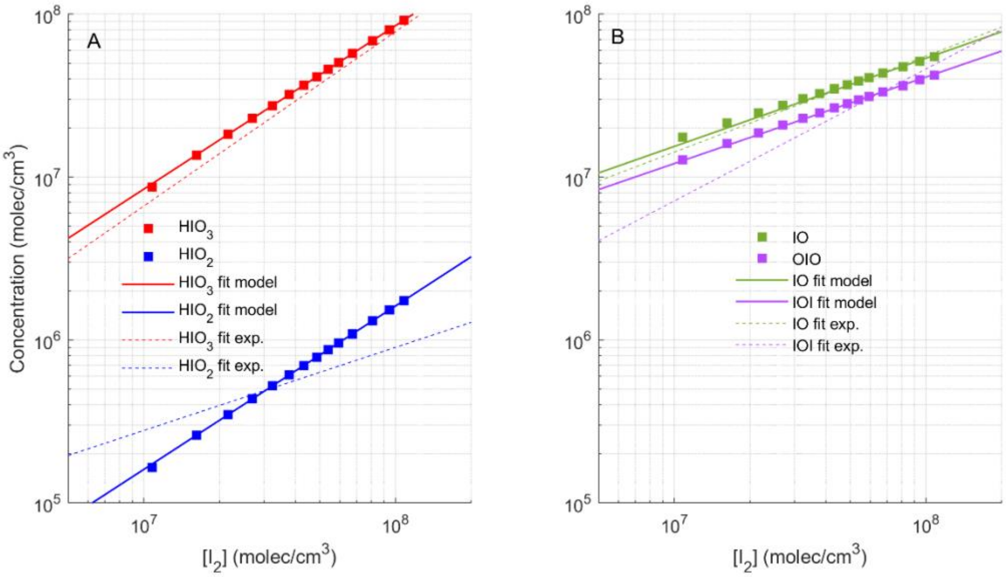

Figure S7. Panel A shows the modelled steady-state HIO_3 and HIO_2 concentrations for conditions resembling the CLOUD chamber experiments presented by He *et al.* (2021) (supplementary figure S2 in He *et al.*). The lines are power-law fits to the HIO_3 and HIO_2 concentrations of the form $[HIO_x]=k\cdot[I_2]^n$, with reported experimentally fitted values for n of 1.07±0.04 and 0.51±0.04, respectively. Based on the model results we fitted values of n equal to 1.00 for both HIO_3 and HIO_2. Panel B shows the modelled steady-state IO and OIO concentrations. The lines are power-law fits to the IO and OIO concentrations of the form $[IO_x]=k\cdot[I_2]^n$, with reported experimentally fitted values for n of 0.59±0.03 and 0.81±0.02, respectively. Based on the model results we fitted values of n for IO and OIO equal to 0.54 and 0.53, respectively.

In the IA-scaling atmospheric simulation, omitting (R1) increases the IOIO concentrations in the simulation which further produces substantially higher concentration of HIO_2 as shown in Figure S8. The *BaseCase* HIO_2 values are lower than the the values observed in ambient coastal regions, where the observed lower limit of $[HIO_2]$ were estimated to be ca. $2x10^6$ cm^{-3} [25]

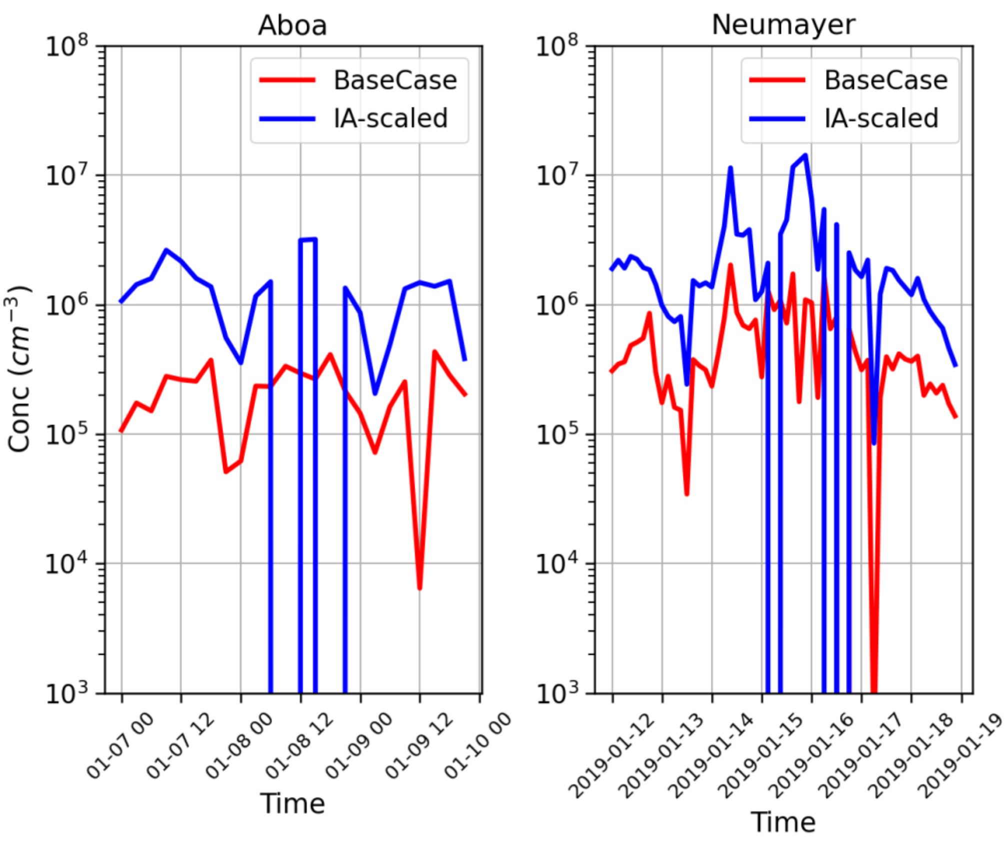

Figure S8. Simulated HIO_2 concentrations for *IA-scaling* and *BaseCase*.

HIO_3 formation from CH_3I

Below we outline a few reactions (**R4**-**R11**) that lead to the formation of HIO_3 form CH_3I. it should be noted that OIO can form HIO_3 via reacting with OH (**R9**) or further react with OIO to form IOIO which further via reaction **R1-R2** forms HIO_3. **R12** indicates the formation of IOIO from inorganic HOI emissions. I_2 ozonolysis and/or reaction with OH (R13-R14) can also eventually form IOIO via formation of HOI, IO and OIO pathways (R8-R12).

$CH_3I + OH \rightarrow CH_3IO_2$; $k=4.3 \times 10^{-12}(\exp^{(-1120/T)})$ **(R4)**

$CH_2IO_2 + HO_2 = CHOI$; $k=0.15 \times 6.7 \times 10^{-12}$ **(R5)**

$CHOI + OH = I + CO$: $k=1.16 \times 10^{-12}$ **(R6)**

$I + O_3 = IO$; $2.1 \times 10^{-11} (\exp^{(-830/T)})$ **(R7)**

$IO + IO = OIO + I$; $0.38 \times 8.03 \times 10^{-11}(\exp^{(500*(1/T-1D0/298D0))})$ **(R8)**

$OIO + OH = HIO_3$; $k= 0.5 \times 2 \times 10^{-10}$ **(R9)**

$OIO + OIO = IOIO$; $k=5 \times 10^{-11}$ **(R10)**

$IO + IO = IOIO$; $k=0.485 \times 8.03 \times 10^{-11}(\exp^{(500*(1/T-1D0/298D0))})$ **(R11)**

$HOI + OH = IO$; $k= 2 \times 10^{-13}$ **(R12)**

$I_2 + OH = I + HOI$; $k=2.1 \times 10^{-10}$ **(R13)**

$I_2 + O_3 = IO + OIO$: $k= 0.5 \times 4 \times 10^{-15}(\exp^{(-2050/T)})$. **(R14)**

All reaction rate constants are in (cm^3 $molec^{-1}$ s^{-1}). IO once formed via **R12** can eventually form IOIO via either **R11** or **R8-R10**.

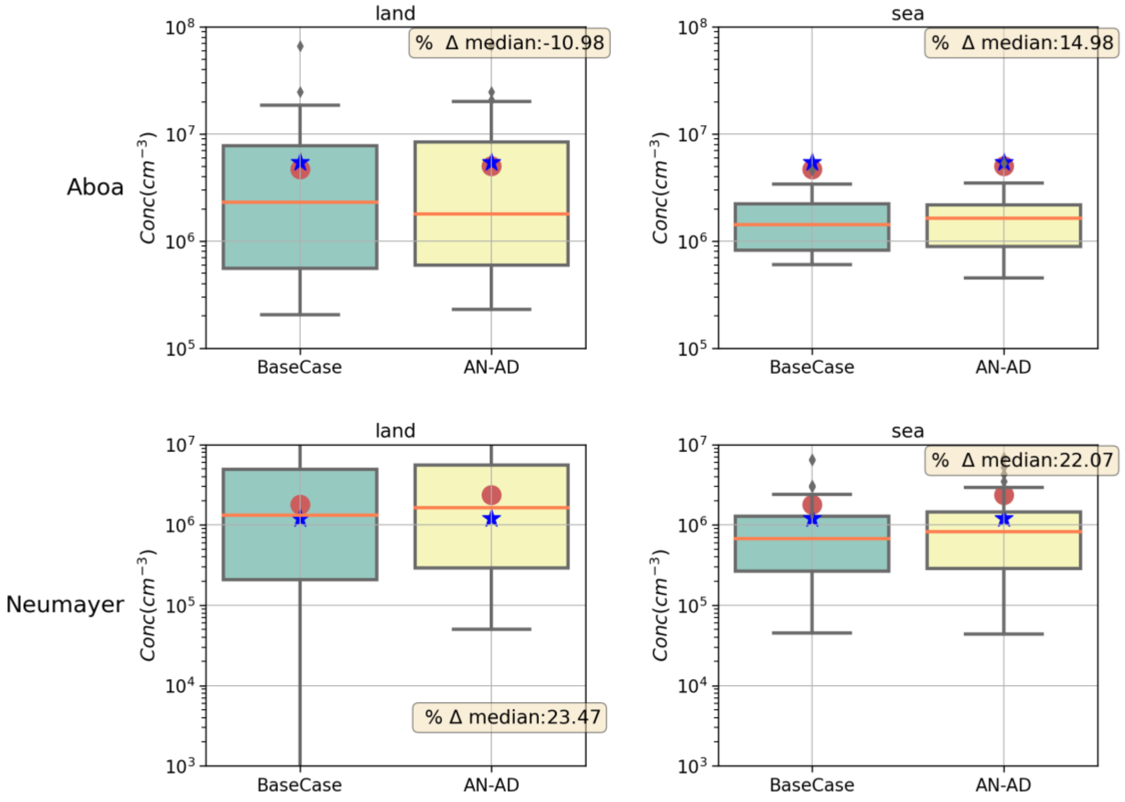

Figure S9. Simulated SA gas-phase concentrations over land and sea for Aboa (upper panel) and Neumayer (lower panel) for both the *BaseCase* and *AN-AD* simulations. The central coral colored line in the box represents median values while the whiskers indicate the maximum and minimum values. The % Δ median values indicate the relative change in SA(g) concentration i.e ($SA_{(AN\text{-}AD)} - SA_{(BaseCase)})/SA_{(BaseCase)}$. The red points in each figure represent the simulated median SA at the station for reference (Table S3). The blue points indicate the median measured gas-phase concentrations at the station for reference.

Table S3: Measured and simulated median SA gas concentrations at the station for the *AN-AD* and *BaseCase*.

**Station**		**Simulated (10^6 #cm^{-3})**	**Measured (10^6 #cm^{-3})**
**Aboa**	**AN-AD**	3.94	5.44
	**BaseCase**	3.21	5.44
**Neumayer**	**AN-AD**	2.02	1.21
	**BaseCase**	1.56	1.21

The lower relative median SA(g) for the *BaseCase* simulation in comparison to *AN-AD* case, can indicate the SA is reduced by condensation onto the IA-HIO_2 particles, resulting in less SA available to cluster with NH_3 or DMA. The % Δ median values give an estimation of scavenging by particles.

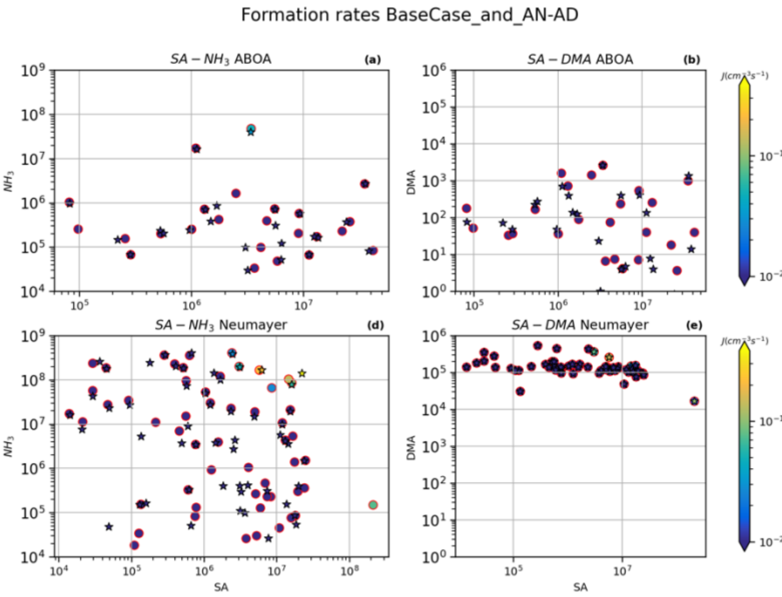

Figure S10. Formation rates (J_{nucl}, cm^{-3} s^{-1}) at Aboa and Neumayer stations via the SA-NH_3 (**a** and **d**), SA-DMA (**b** and **e**) pathways for both the *BaseCase* (star) and the *AN-AD* (circle) simulations.

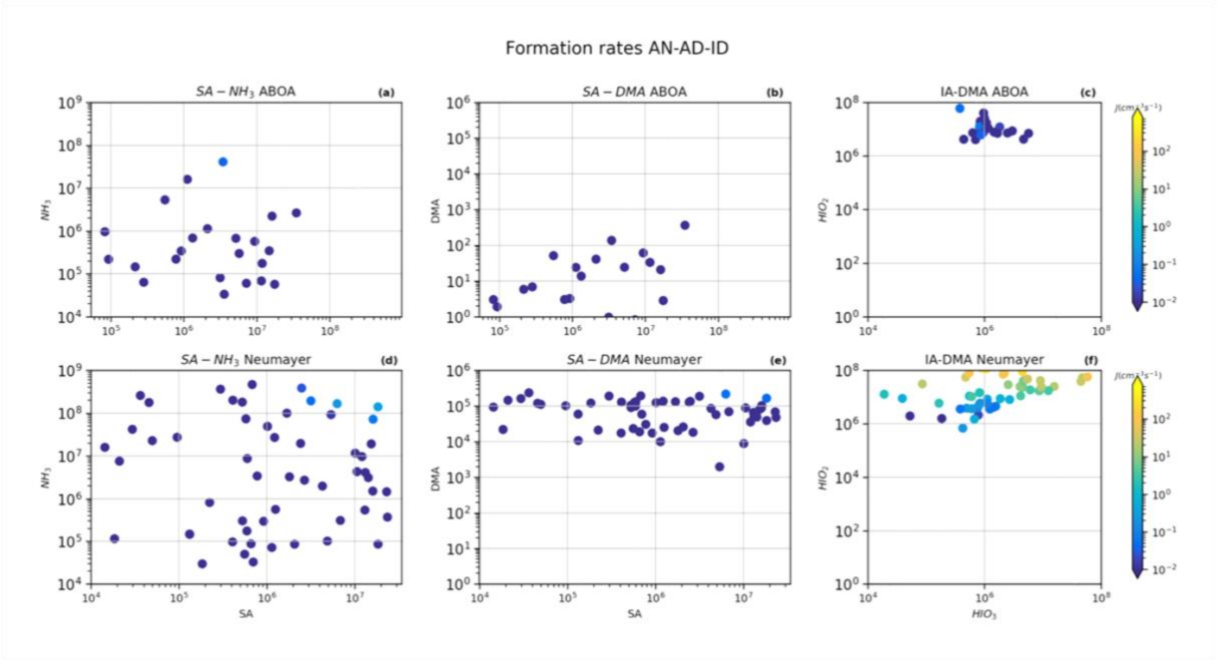

Figure S11. Formation rates (J_{nucl}, cm^{-3} s^{-1}) at Aboa and Neumayer stations via the SA-NH_3 (**a** and **d**), SA-DMA (**b** and **e**) and IA-DMA (**c** and **f**) pathways for the *AN-AD-ID* simulations.

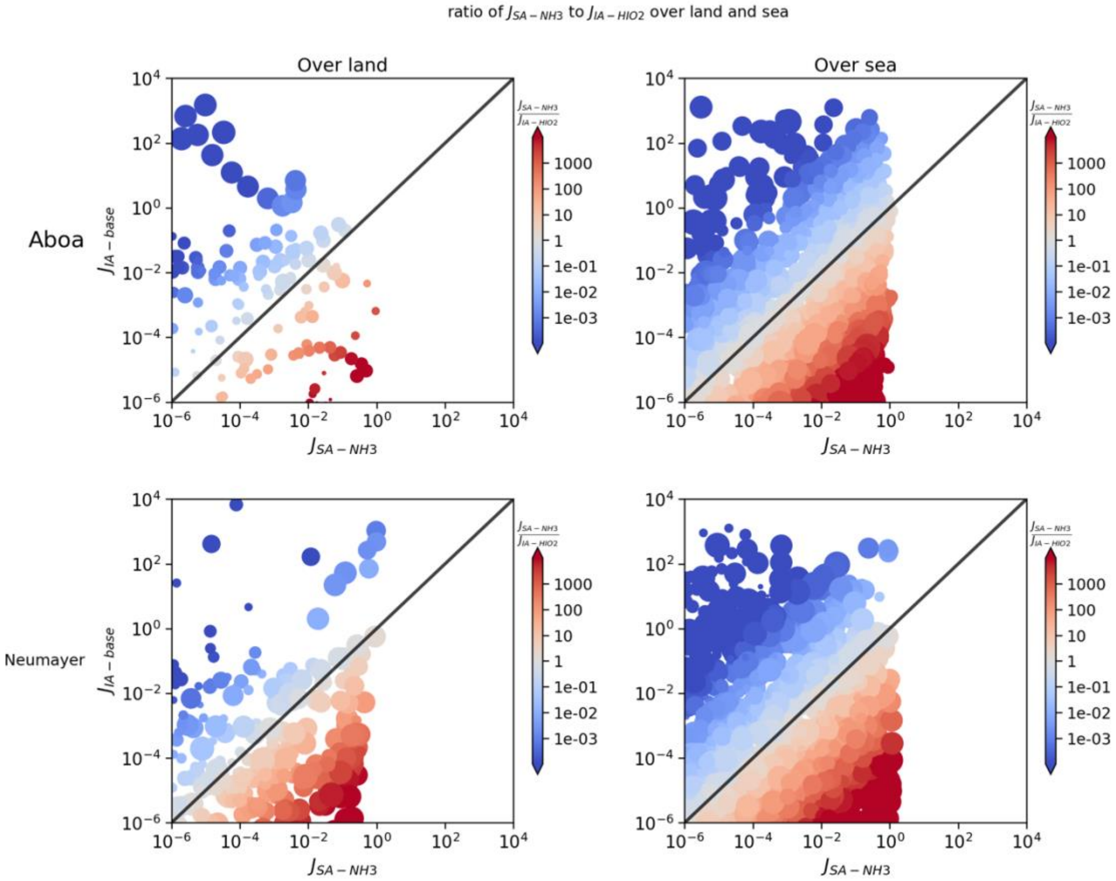

Figure S12. Ratio of formation rates $J_{SA-NH3}/J_{IA-HIO2}$ for air masses over land and sea arriving at both Aboa and Neumayer stations. The size of the markers indicate how far they are from the station, with smaller points representing airmasses closer to the station and larger points representing airmasses further away. Only J values greater than 10^{-6} cm^{-3} s^{-1} are shown in the figure. The black line indicates 1:1 ratio of x and y values.

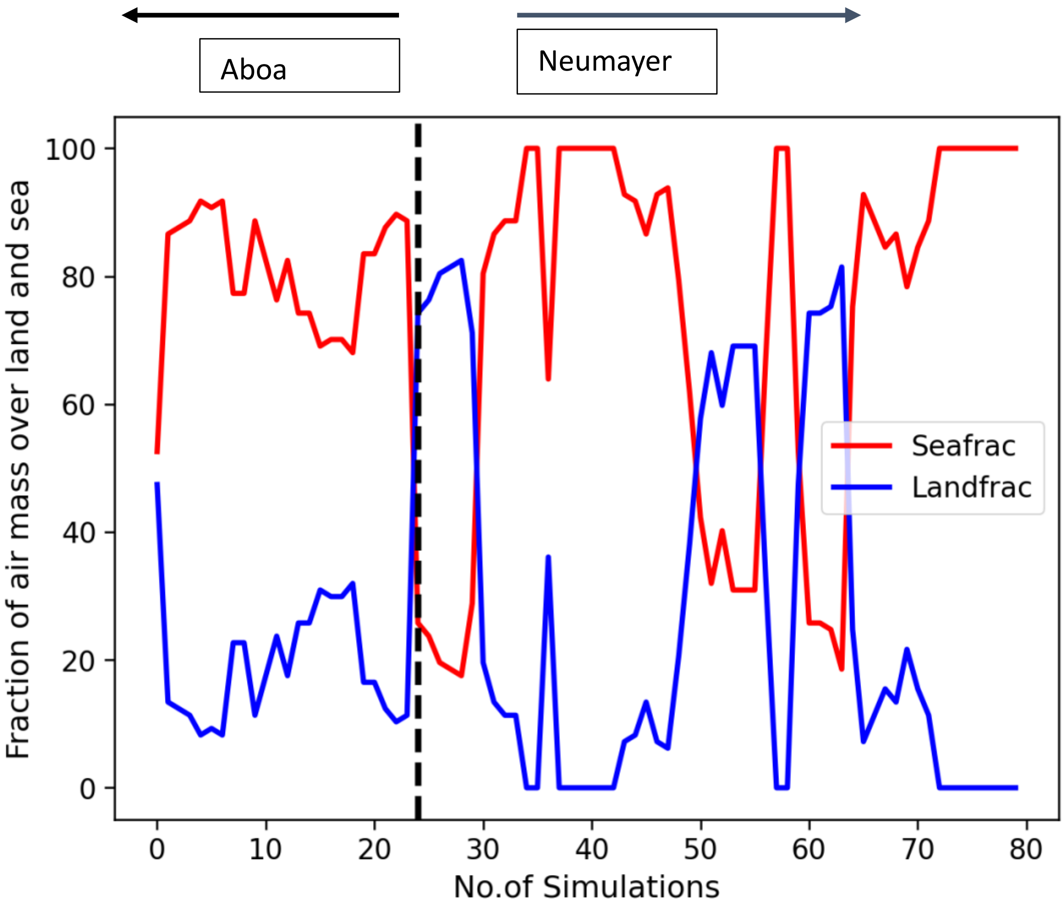

Figure S13. The fraction of time the air mass has spent over either land or sea for each simulation. Simulations number 0-23 are for Aboa and 24-80 are for Neumayer.

The surface is now classified as land if the land categorization in the model is either land or sea ice or snow with either zero or very low DMS concentrations (< 10^{-15} kg $m^{-2}s^{-1}$) since sea ice can potentially emit DMS[26].

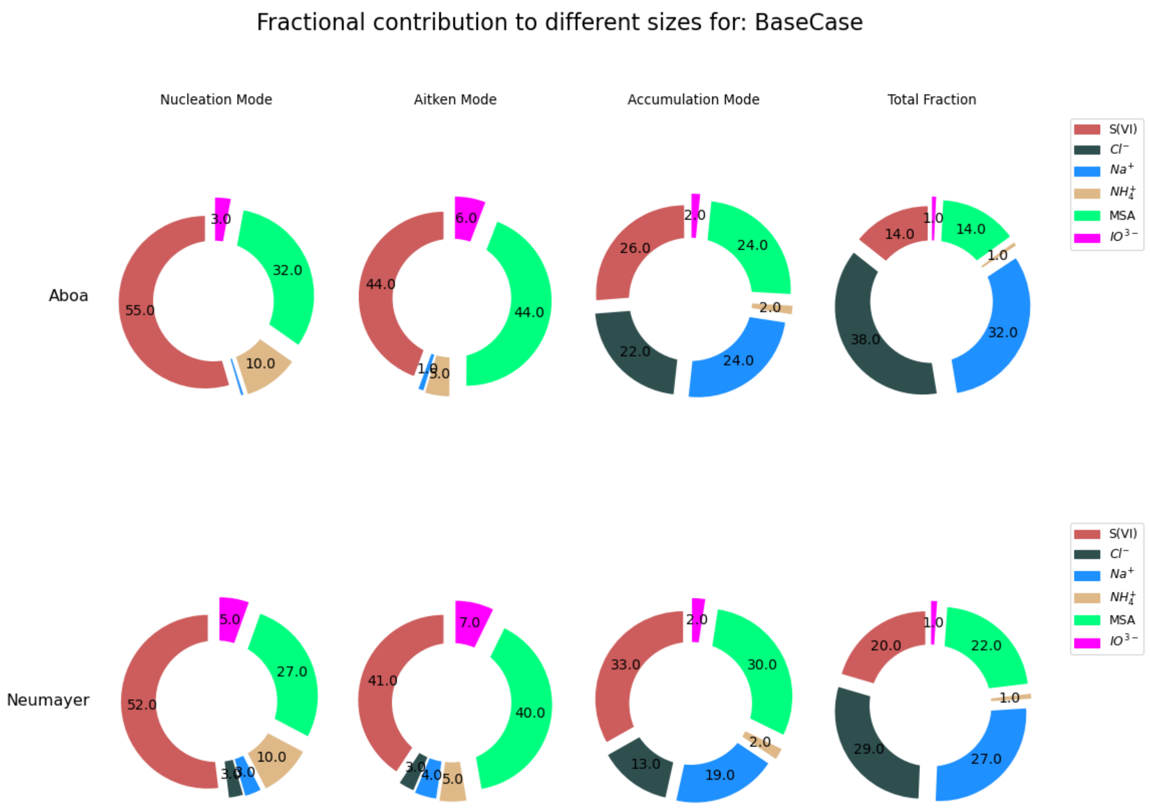

Figure S14. Normalized contribution of different species in different size modes (nucleation, Aitken, accumulation, and total (1 nm-10 μm)) at both Aboa (upper panel) and Neumayer (lower panels). All the values are rounded to the nearest digit. Normalized concentration is a calculated based on the maximum concentration in every mode.

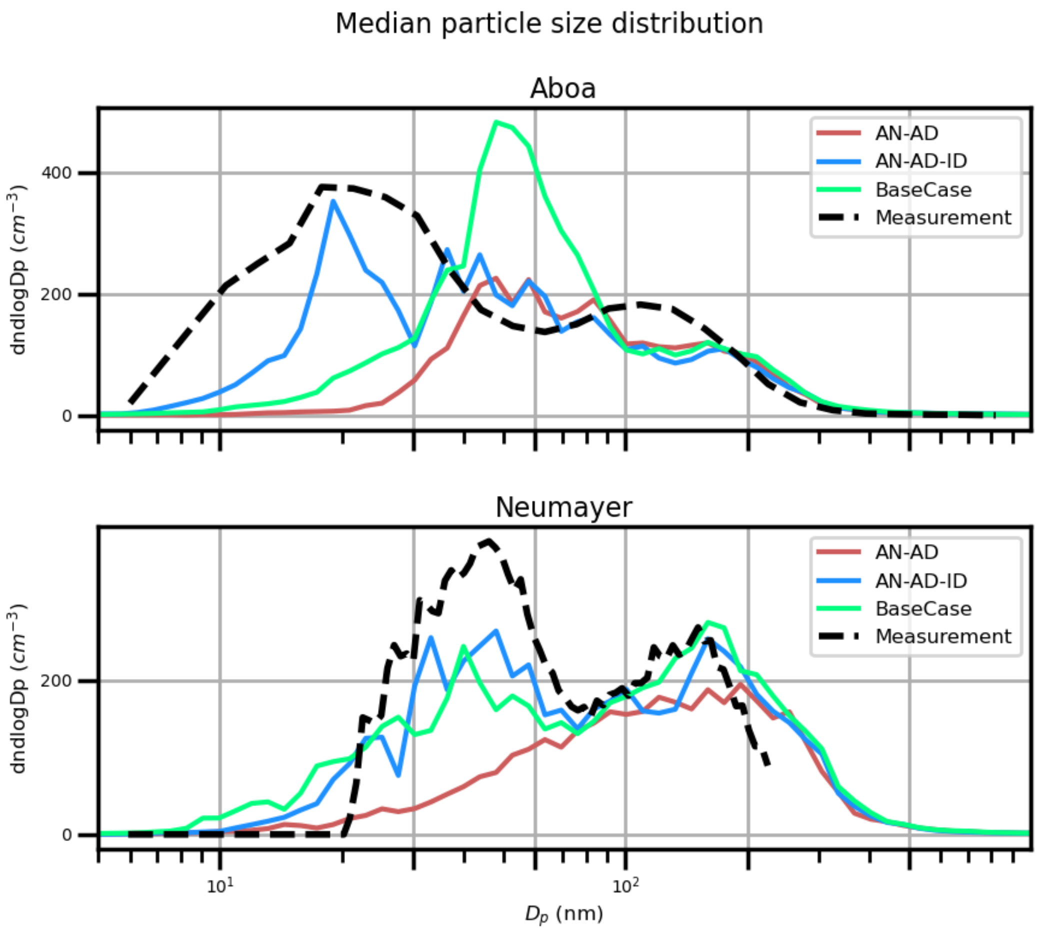

Figure S15. Median particle number size distributions at Aboa (upper panel) and Neumayer (lower panel) for the *BaseCase, AN-AD and AN-AD-ID* simulations, compared with ambient measurements.

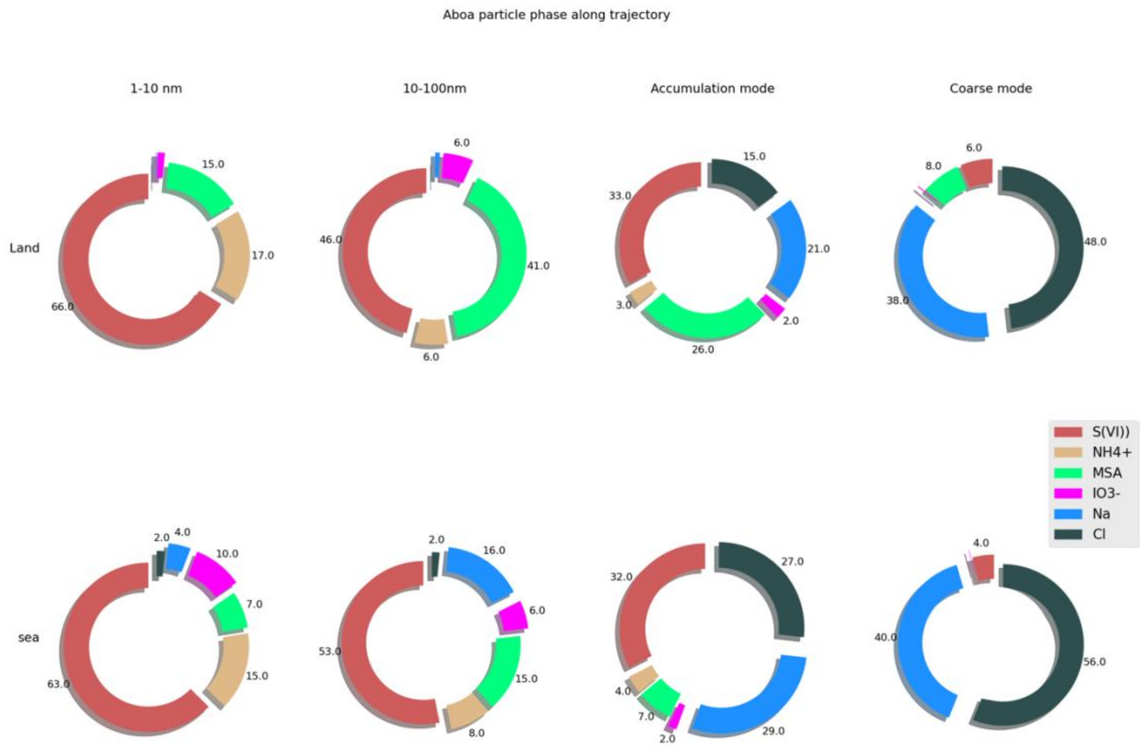

Figure S16. Normalized particle phase mass fractions for air masses arriving at Aboa for the *BaseCase* simulation. The upper panel depicts the mass fraction when the airmass spends time over land and the lower panel indicates the mass fraction when the air mass spends time over sea. The nucleation mode in this figure indicates particles between 1-10 nm, Aitken mode corresponds to 10-100 nm, while accumulation and coarse mode size ranges are same as before. The nucleation mode in this figure only includes particles between 1-10 nm to analyze the contribution of different clustering systems in NPF. Normalized concentration is a calculated based on the maximum concentration in every mode for land and sea.

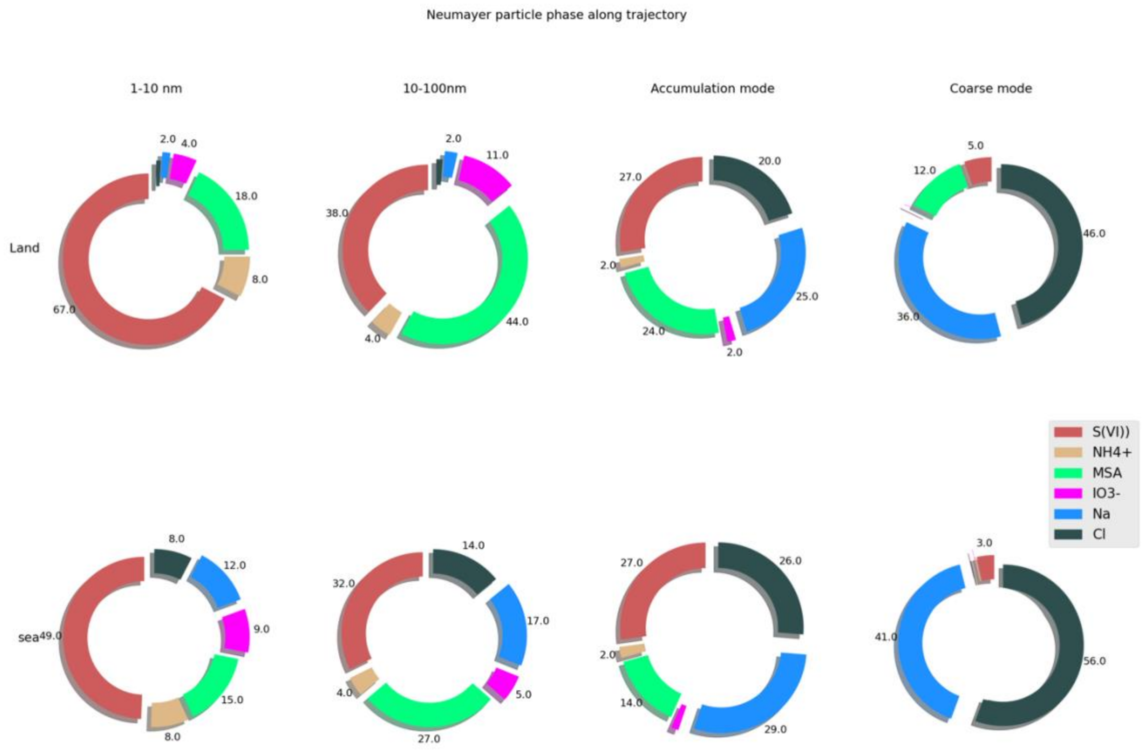

Figure S17. Normalized particle phase mass fractions for air masses arriving at Neumayer for the *BaseCase* simulation. The upper panel depicts the mass fraction when the airmasses spends time over land and the lower panel indicates the mass fraction when the air mass spends time over sea. The nucleation mode in this figure indicates particles between 1-10 nm, Aitken mode corresponds to 10-100nm, while accumulation and coarse mode size ranges are same as before. The nucleation mode in this figure only include particle between 1-10 nm to analyze the contribution of different clustering systems in NPF. Normalized concentration is a calculated based on the maximum concentration in every mode for land and sea.

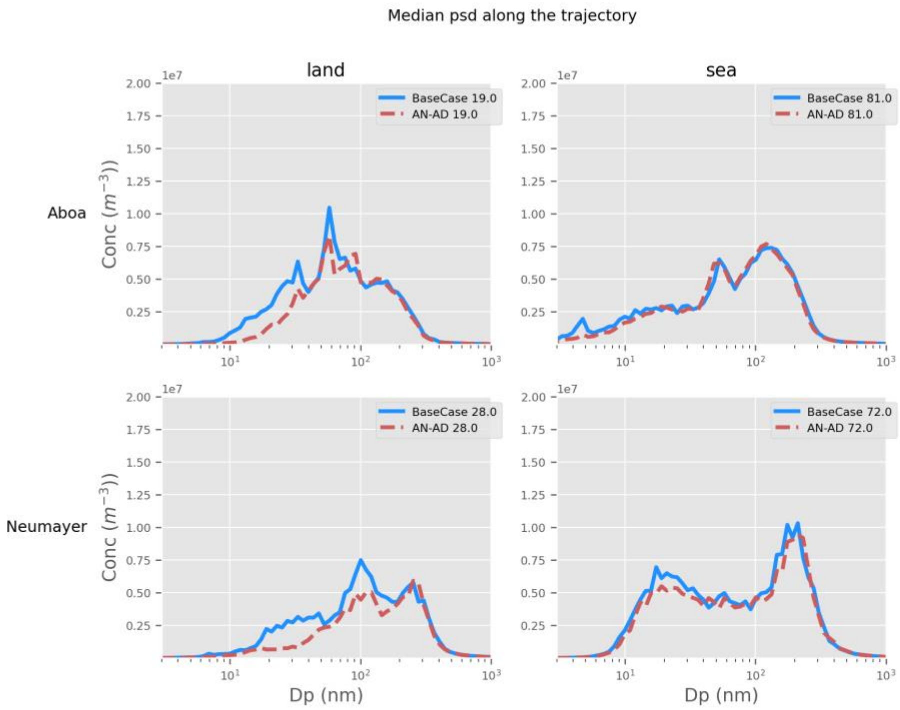

Figure S18. Median particle number size distributions over land and sea air masses arriving at Aboa (upper panel) and Neumayer (lower panel) for the *BaseCase* and *AN-AD* simulation. The legend values represent the average time spent by the air mass over land and sea. The measurement data has not been included in this figure, since this figure represents the particle number size distribution along the trajectory, just before the air mass arrives at the station.

Figure S18 shows higher particle number concentrations below 10 nm over sea in the airmasses arriving at Aboa, while over land the particle number size distribution is shifted towards larger sizes (10 – 70 nm). This suggests that NPF and early growth predominantly happens over sea while the growth of the small particles to Aitken mode happens over land. The model shows a similar behavior at Neumayer wherein the NPF and initial growth generally happens over the open seas as compared to land.

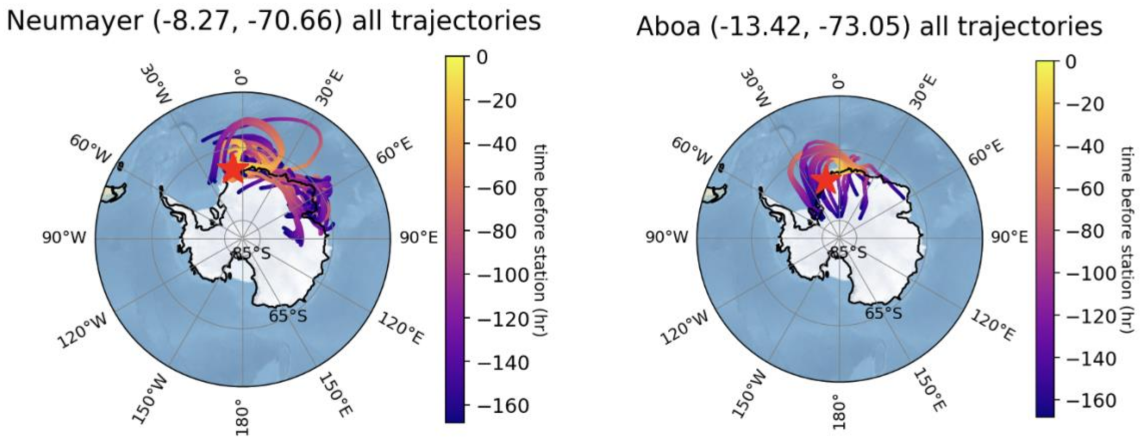

Figure S19. The left and right panels show all the mean trajectories arriving at Neumayer and Aboa during the simulation period. The trajectories are color coded with respect to the time of arrival at the station with 0 hr indicating time at the station and -168 indicating 7 days back. The red stars indicate the stations.

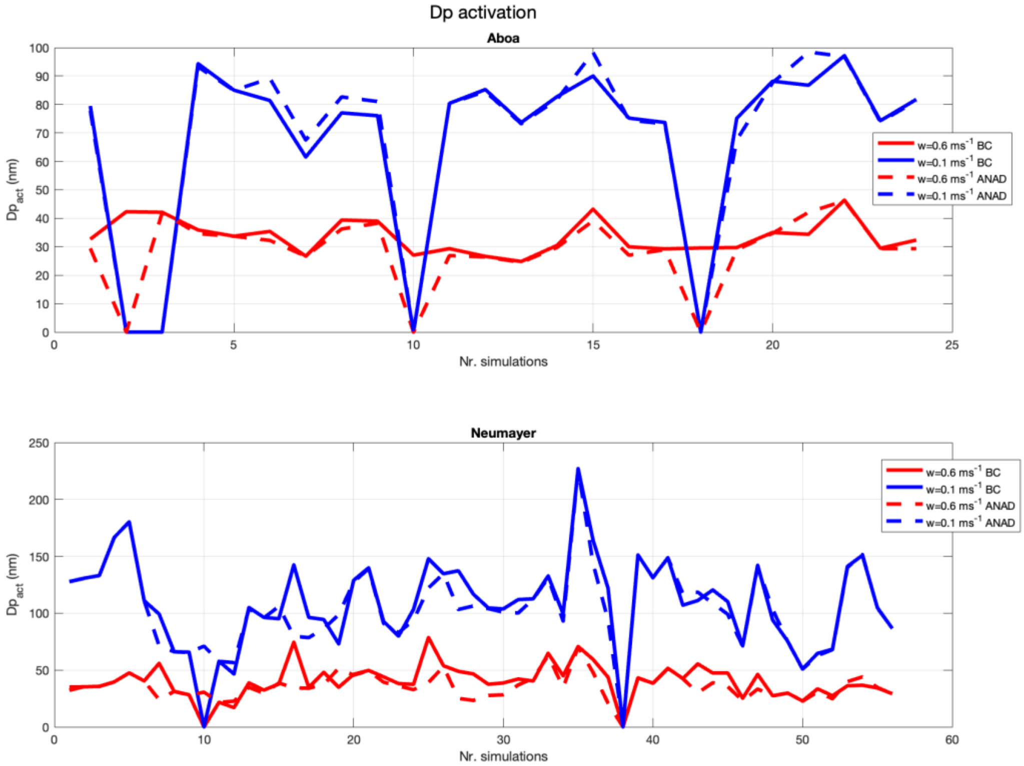

Figure S20: The upper panel shows the simulated activation diameter (Dp_{act}) at Aboa and the lower panel shows the activation diameter at Neumayer. These results are derived from the adiabatic cloud parcel model.

The median activation diameter at Aboa and Neumayer is ~78 and ~80 nm respectively.

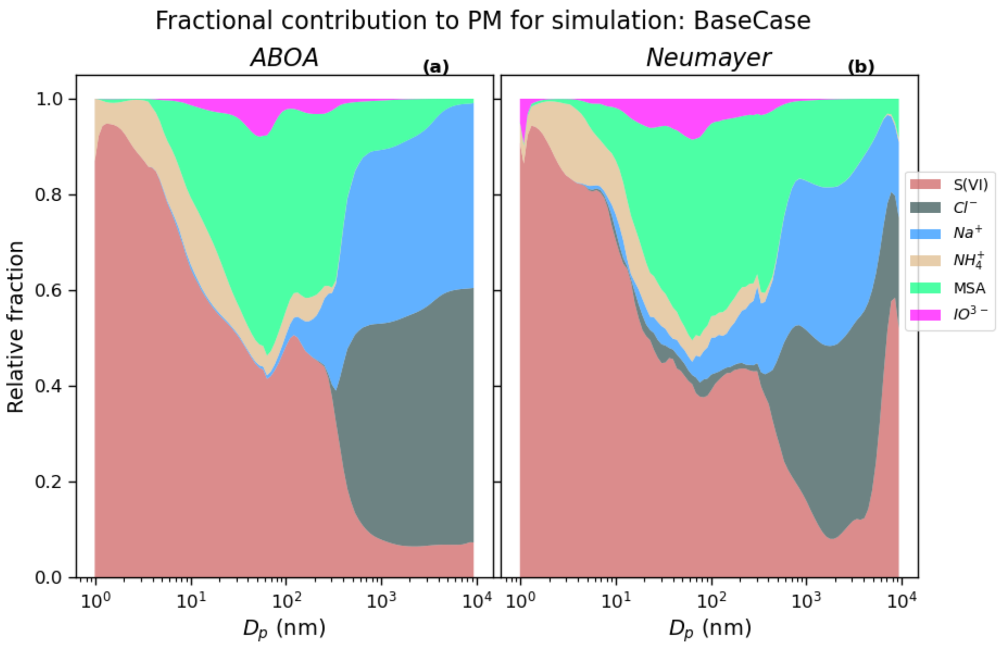

Figure S21. Particle phase relative fractions for different species at different at Aboa (**a**) and Neumayer (**b**). The particle mass relative fractions in the figure are indicative of the species which contribute to the growth and survival of the particles measured at the stations.

Table S4: Median CCN number concentration changes (ΔCCN) due to the iodine-assisted particle formation, relative to *AN-AD* simulation for 3 updraft velocities (*w*=0.1, 0.6 and 1.0 m s^{-1}). The median SS for CCN activation and 25th-75th percentile values at different *w* is shown in grey shaded rows.

	***w*=0.1 m s^{-1}**	***w*=0.6 m s^{-1}**	***w*=1.0 m s^{-1}**
**Aboa ΔCCN (%)**	-4.90	101.13	114.34
SS_{Aboa} (%, 25th-75th percentile)	0.15 (0.13-0-18)	0.67 (0.56-0.76)	1.22 (1.06-1.35)
**Neumayer ΔCCN (%)**	10.67	21.34	37.44
SS_{Neu} (% 25th-75th percentile)	0.1 (0.09-0.12)	0.43 (0.36-0.55)	0.84 (0.75-1.03)

References

1. Roldin, P. *et al.* The role of highly oxygenated organic molecules in the Boreal aerosol-cloud-climate system. *Nat Commun* **10**, 4370 (2019).
2. Xavier, C. *et al.* Secondary aerosol formation in marine Arctic environments: a model measurement comparison at Ny-Ålesund. *Atmos Chem Phys* **22**, 10023–10043 (2022).
3. Pisso, I. *et al.* The Lagrangian particle dispersion model FLEXPART version 10.4. *Geosci Model Dev* **12**, 4955–4997 (2019).
4. Stohl, A., Forster, C., Frank, A., Seibert, P. & Wotawa, G. Technical note: The Lagrangian particle dispersion model FLEXPART version 6.2. *Atmos Chem Phys* **5**, 2461–2474 (2005).
5. Granier, C. *et al.* The Copernicus Atmosphere Monitoring Service global and regional emissions (April 2019 version). (2019).

6. Ziska, F. *et al.* Global sea-to-air flux climatology for bromoform, dibromomethane and methyl iodide. *Atmos Chem Phys* **13**, 8915–8934 (2013).
7. Sofiev, M., Soares, J., Prank, M., de Leeuw, G. & Kukkonen, J. A regional-to-global model of emission and transport of sea salt particles in the atmosphere. *Journal of Geophysical Research: Atmospheres* **116**, (2011).
8. Barthel, S., Tegen, I. & Wolke, R. Do new sea spray aerosol source functions improve the results of a regional aerosol model? *Atmos Environ* **198**, 265–278 (2019).
9. Riddick, S. N. *et al.* The global distribution of ammonia emissions from seabird colonies. *Atmos Environ* **55**, 319–327 (2012).
10. Carpenter, L. J., Archer, S. D. & Beale, R. Ocean-atmosphere trace gas exchange. *Chem Soc Rev* **41**, 6473–6506 (2012).
11. Besel, V., Kubečka, J., Kurtén, T. & Vehkamäki, H. Impact of Quantum Chemistry Parameter Choices and Cluster Distribution Model Settings on Modeled Atmospheric Particle Formation Rates. *Journal of Physical Chemistry A* **124**, 5931–5943 (2020).
12. Myllys, N. *et al.* Role of base strength, cluster structure and charge in sulfuric-acid-driven particle formation. *Atmos Chem Phys* **19**, 9753–9768 (2019).
13. Zhang, R. *et al.* Critical Role of Iodous Acid in Neutral Iodine Oxoacid Nucleation. *Environ Sci Technol* **56**, 14166–14177 (2022).
14. Ning, A. *et al.* The critical role of dimethylamine in the rapid formation of iodic acid particles in marine areas. *NPJ Clim Atmos Sci* **5**, (2022).
15. Olenius, T., Kupiainen-Määttä, O., Ortega, I. K., Kurtén, T. & Vehkamäki, H. Free energy barrier in the growth of sulfuric acid–ammonia and sulfuric acid–dimethylamine clusters. *J Chem Phys* **139**, 084312 (2013).
16. Olenius, T. & Roldin, P. Role of gas–molecular cluster–aerosol dynamics in atmospheric new-particle formation. *Sci Rep* **12**, 1–13 (2022).
17. Myllys, N. *et al.* Role of base strength, cluster structure and charge in sulfuric-acid-driven particle formation. *Atmos Chem Phys* **19**, 9753–9768 (2019).
18. Jokinen, T. *et al.* Atmospheric sulphuric acid and neutral cluster measurements using CI-APi-TOF. *Atmos Chem Phys* **12**, 4117–4125 (2012).
19. Mirme, S. & Mirme, A. The mathematical principles and design of the NAIS - A spectrometer for the measurement of cluster ion and nanometer aerosol size distributions. *Atmos Meas Tech* **6**, 1061–1071 (2013).
20. Wang, S. C. & Flagan, R. C. Scanning Electrical Mobility Spectrometer. *Aerosol Science and Technology* **13**, 230–240 (1990).
21. Finkenzeller, H. *et al.* The gas-phase formation mechanism of iodic acid as an atmospheric aerosol source. *Nat Chem* (2022) doi:10.1038/s41557-022-01067-z.
22. Roldin, P. *et al.* Modelling non-equilibrium secondary organic aerosol formation and evaporation with the aerosol dynamics, gas- and particle-phase chemistry kinetic multilayer model ADCHAM. *Atmos Chem Phys* **14**, 7953–7993 (2014).
23. Stolzenburg, D. *et al.* Enhanced growth rate of atmospheric particles from sulfuric acid. *Atmos Chem Phys* **20**, 7359–7372 (2020).
24. He, X. *et al.* Role of iodine oxoacids in atmospheric aerosol nucleation. **595**, 589–595 (2021).
25. Sipilä, M. *et al.* Molecular-scale evidence of aerosol particle formation via sequential addition of HIO3. *Nature* **537**, 532–534 (2016).

26. Jang, E. *et al.* First-year sea ice leads to an increase in dimethyl sulfide-induced particle formation in the Antarctic Peninsula. *Science of the Total Environment* **803**, (2022).
